# Restricted Open-Shell Hartree–Fock Method for a General Configuration State Function Featuring Arbitrarily Complex Spin-Couplings

**DOI:** 10.1021/acs.jpca.4c00688

**Published:** 2024-06-17

**Authors:** Tiago Leyser da Costa Gouveia, Dimitrios Maganas, Frank Neese

**Affiliations:** Max-Planck-Institut für Kohlenforschung, Kaiser-Wilhelm-Platz 1, Mülheim an der Ruhr 45470, Germany

## Abstract

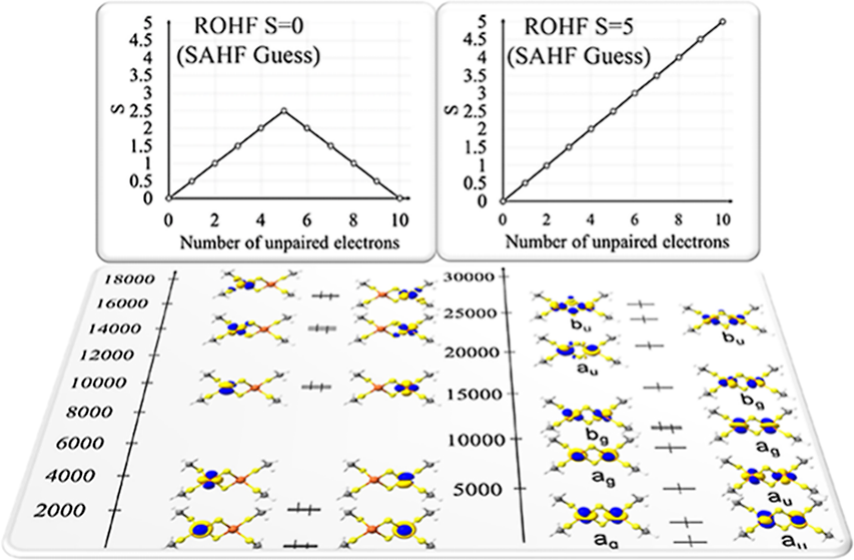

In this work, we present a general spin restricted open-shell Hartree–Fock (ROHF) implementation that is able to generate self-consistent field (SCF) wave functions for an arbitrary configuration state function (CSF). These CSFs can contain an arbitrary number of unpaired electrons in arbitrary spin-couplings. The resulting method is named CSF-ROHF. We demonstrate that starting from the ROHF energy expression, for example, the one given by Edwards and Zerner, it is possible to obtain the values of the ROHF vector-coupling coefficients by setting up an open-shell for each group of consecutive parallel-coupled spins dictated by the unique spin-coupling pattern of any given CSF. To achieve this important and nontrivial goal, we employ the machinery of the iterative configuration expansion configuration interaction (ICE-CI) method, which is able to tackle general CI problems on the basis of spin-adapted CSFs. This development allows for the efficient generation of SCF spin-eigenfunctions for systems with complex spin-coupling patterns, such as polymetallic chains and metal clusters, while maintaining SCF scaling with system size (quadratic or less, depending on the specific algorithm and approximations chosen).

## 1 Introduction

The realization that the majority of “life”-related chemical processes in the fields of (bio)inorganic chemistry, materials science, and catalysis involve multimetallic open-shell chemical systems with electronic ground and excited states possessing complex spin-coupling situations has imposed great challenges to both experimental and theoretical chemistry.^1−12^ The electronic states of these systems are usually characterized by a complex spin alignment of the open-shell electrons, leading to various ferromagnetic or antiferromagnetic-coupled states. The nature of these states is difficult to interpret from the experimental point of view, due to the high density of many-particle states that may be probed in the various experimental techniques^13^ or the strong dependence of the experimental measurements to the actual experimental conditions, often leading to dependencies (e.g., temperature, field, and radiation) that may be difficult to control or interpret.

The theoretical characterization of such systems is also challenging due to the computational complexity associated with the description of the involved ground and excited many-particle states. The most common practice is to break the spin symmetry of the system and perform mean-field level computations over chosen broken-symmetry Slater determinants on the basis of a variational orbital optimization (the self-consistent field (SCF)-procedure). In conjunction with density functional theory (BS-DFT), this constitutes the most commonly used approach in tackling systems with complex spin-couplings.^14−25^ However, it should be emphasized that BS-DFT gives only a crude representation of antiferromagnetic couplings, as it is unable to correctly describe the electron correlation phenomena in these systems.

In fact, problems of such complexity require elaborate multiconfigurational wave function-based methods. In particular, the complete active space SCF (CASSCF) method is a well-established approach in quantum chemistry for the treatment of strongly correlated electron systems with substantial multireference character.^26−31^ Recent advances of approximate configuration interaction (CI) wave function-based methods in the framework of the density matrix renormalization group (DMRG),^32^ the full configuration interaction quantum Monte Carlo (FCIQMC),^33−35^ or selected CI approaches,^36,37^ such as the iterative configuration expansion CI (ICE-CI),^38,39^ for treating large active spaces have helped to drastically reduce the size limitations of the conventional CAS-based approaches, to the chemical sensible active space that needs to be correlated in order to probe the couples spin-coupling problem of several systems.

In most, if not all, of these electronic structure calculations, the initial step is a mean-field Hartree–Fock (HF) calculation, which in the context of open-shell systems might face severe convergence problems. In practice, it is quite common that for open-shell systems, the starting orbitals are obtained from the solution of a high spin (HS)-restricted open-shell HF (ROHF) calculation. The ROHF method was first described by Roothan.^40^ Differently from the more popular unrestricted HF (UHF) method, the ROHF wave function is restricted to be a spin-eigenfunction, which makes it well suited for posterior post HF wave function-based calculations. In principle, one can consider ROHF methods to be specific cases of multiconfiguration SCF (MCSCF) methods. In both cases, the wave function consists of a linear combination of Slater determinants. However, in ROHF, the coefficients of the linear expansion are chosen to represent a particular spin state and/or symmetry and are not allowed to vary freely, as in MCSCF.

The most common formulation of the ROHF problem is for the HS case, where the open-shell structure consists of only parallel-coupled spins. However, several other formulations for treating other spin-coupling situations exist in the literature, such as configuration-averaged HF (CAHF)^41^ and spin-averaged HF (SAHF),^42^ among others.^43,44^ Together with the improvement in numerical methods for computing ROHF, they all have helped to overcome the convergence problems of ROHF and popularize it in the quantum chemistry community. Although several low spin (LS) ROHF cases have been formulated, to the best of our knowledge, a rigorous configuration state function (CSF)-based formulation for arbitrary spin-coupling situations has never been reported. This seems to be of paramount importance as it has been shown that approximate CI methods employed on HS ROHF orbitals perform very poorly in describing other spin-coupling situations.^35^

Hence, in this paper, we present a new procedure to set up ROHF calculations of a single CSF with arbitrarily complex spin-couplings. Thus, we have made use of the general infrastructure of the recently developed CSF-based ICE algorithm that allows the generation of any desired CSF on the fly for subsequent solving of the respective ROHF problem. We refer to the resulting methods as CSF-ROHF. After presenting the theory and demonstrating its performance over the conventional CASSCF in a Ni(II) chain with increasing number of nickel atoms, we demonstrate the ability of the CSF-ROHF method to probe a series of solutions of the Hund and non-Hund CSFs of the [Fe(SCH_3_)_4_]^−^ complex, the ferromagnetic and antiferromagnetic states of the [Fe_2_S_2_(SCH_3_)_4_]^2–^ and [Gd_2_Cl_11_]^5–^ dimers, as well as to probe the experimentally accepted ground state spin-coupling situations on the trimer [Cu_3_(OH)_3_(en)_3_]^3+^, the cubane [Fe_4_S_4_(SCH_3_)_4_]^2–^, and the complex [Co(^1^L_N_)_2_], ^1^L_N_ = C_6_H_4_(NH_2_).

## 2 Theory

### 2.1 General CSF-Based ROHF Formulation

We start by recalling the ROHF energy expression 1 given by Edwards and Zerner^45^
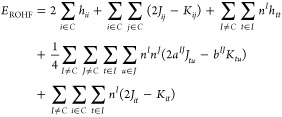
1where *h*_pq_ is the one-electron integral (possibly containing contributions from external point charges or relativistic corrections), *J*_pq_ and *K*_pq_ are the Coulomb and exchange integrals, respectively, *n*^*I*^ is the occupation number of shell *I*, *C* being the closed-shell, and *a*^*IJ*^ and *b*^*IJ*^ are the so-called vector-coupling coefficients. In the following text, we use the indices *i*, *j*, *k*, and *l* for doubly occupied molecular orbitals, *t*, *u*, *v*, and *w* for singly occupied molecular orbitals (SOMOs), and *p*, *q*, *r*, and *s* for generic molecular orbitals.

In contrast to methods like CAHF or SAHF that average over configurations and therefore typically feature fractional occupation numbers, the CSF method constrains the occupations to *n*^*I*^ = 1. The spin-coupling situation of the unpaired electrons is determined by the number of open-shells and the vector-coupling coefficients. Hence, for setting up the ROHF problem for a given CSF, we need to determine the values of *a*^*IJ*^ and *b*^*IJ*^ that appropriately describe it.

We can achieve this by constructing the CSFs using one of the many available construction methods for spin-eigenfunctions,^46^ for example, the genealogical spin-coupling scheme of Grabenstetter et al.^47^ In this scheme, a given CSF is built by sequentially adding the electrons in such a way that the spin-coupling information on an electron *u* in a SOMO is specified with respect to all other SOMOs *t* < *u*.

The expectation value of the Born–Oppenheimer Hamiltonian in the second quantization for a given CSF is then given by eq 2
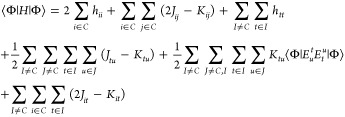
2

The matrix element ⟨Φ|*E*_*u*_^*t*^*E*_*t*_^*u*^|Φ⟩ can be evaluated directly, without expanding the CSF into a linear combination of Slater determinants, as explained elsewhere^48^ and as applied in the context of the ICE-CI^38,39^ present in ORCA.^49−53^ Being able to directly evaluate the ⟨Φ|*E*_*u*_^*t*^*E*_*t*_^*u*^|Φ⟩ element, the value of the vector-coupling coefficients can be obtained as follows:

By comparing the terms involving only the open shells of eq 2 with the open-shell terms of the ROHF energy expression 1 for *n*^*I*^ = 1, one arrives on eq 3
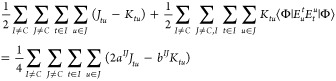
3

Solving the above expression for *a*^*IJ*^ and *b*^*IJ*^, the relations, eqs 4–6 are obtained
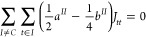
4
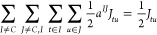
5
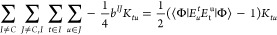
6

From these, we arrive at the following general formulas for the vector-coupling coefficients
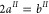
7
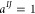
8
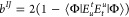
9

This way, the determination of the *b*^*IJ*^ vector-coupling coefficient reduces to the evaluation of the matrix element ⟨Φ|*E*_*u*_^*t*^*E*_*t*_^*u*^|Φ⟩ for the CSF in which the ROHF procedure is to be set. We note here that all t,u orbitals within a given open-shell will produce the same value from the ⟨Φ|*E*_*u*_^*t*^*E*_*t*_^*u*^|Φ⟩ matrix element. The vector-coupling coefficients within a given shell *a*^*II*^ and *b*^*II*^ are related only by eq 7 and can be defined in any way that satisfies it.^45^

It is now possible to set up the Fock operator of the open-shells of the system. In the implementation of the general ROHF method available in the ORCA program, we employ the Rico-Fernandez^54^ formulation of the Fock matrix elements of the open-shells
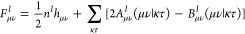
10where *A*_μν_^*I*^ and *B*_μν_^*I*^ are special densities
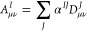
11
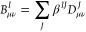
12*D*_μν_^*J*^ is the density matrix element for shell *J*
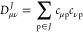
13

While α^*IJ*^ and β^*IJ*^ are related to *a*^*IJ*^ and *b*^*IJ*^ by
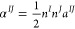
14
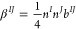
15

The above relations imply that for a given CSF with an arbitrary spin-coupling pattern, it is possible to obtain the values of the ROHF vector-coupling coefficients for a given CSF by setting an open-shell for each group of consecutive parallel-coupled spins.

**Figure 1:**
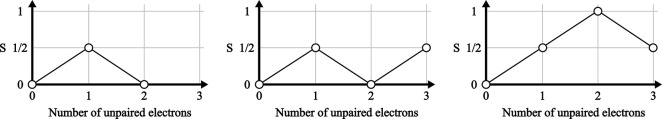
Branching diagrams for the [+ −] (left), [+ + −] (center), and [+ – +] (right) CSFs built from the genealogical-coupling scheme.

As a numerical example, let us consider the cases of CSFs consisting of (a) two unpaired electrons coupling to an open-singlet [+ −] with *S* = 0, and (b) three unpaired electrons coupling to open-doublets (), where two CSFs are possible: [+ – +] and [+ + −], as shown in Figure 1.

As stated above, the open-shells are defined as each set of consecutive parallel-coupled spin. Therefore, the open-singlet [+ −] CSF contains two open-shells and the open-doublets [+ – +] and [+ + −] contain three and two open-shells, respectively.

The values obtained for the open-singlet and the two open-doublet cases are presented in Table 1. We note that the values of the [+ −] and [+ – +] CSFs are in agreement with the ones determined by Edwards and Zerner, which are presented on lines 6 and 8 of Table 1 of ref (45), together with the *b*^*II*^ values of the given cases.

**Table 1:** Coupling Coefficients and Vector-Coupling Coefficients for the Open-Singlet CSF, and the Two Open-Doublet CSFs, as Shown on Figure 1^a^

CSF	number of open-shells	⟨Φ|*E*_*u*_^*t*^ *E*_*t*_^*u*^|Φ⟩	*b*^*IJ*^
+ −	2	2	–2
+ + −	2	1.5	–1
+ – +	3	2, 0.5, 0.5	–2, 1, 1

a Further examples can be found in Table 2.

The procedure outlined above was implemented in a development version of the ORCA computational package and will be part of the next major public release.

### 2.2 Computational Details

All calculations were performed in a development version of the ORCA 6.0 suite of programs.^49−53^

Geometries for the [Fe(SCH_3_)_4_]^−^ and [Fe_2_S_2_(SCH_3_)_4_]^2–^ complexes are obtained from refs (55 and 56,) respectively. For the [Fe_4_S_4_(SCH_3_)_4_]^2–^ cubane system, the geometry used is taken from ref (57). All other geometries were calculated at the DFT level of theory employing the BP86 functional^58,59^ together with Grimme’s dispersion correction^60−64^ with the triple-ζ Def2-TZVP^65^ basis set together with the Def2/J auxiliary basis for the resolution of the identity approximation.^66^ The Ni chain geometry was fixed to local octahedral geometries,^67^ with only the hydrogen atoms being optimized at the same level of theory of the other geometry optimizations. The coordinates of all the geometries used in this paper can be found in the Supporting Information.

CI calculations were performed using the CSF-based version of ICE-CI method.^38,39^ The triple-ζ quality Def2-TZVP^65^ basis set was used for all atoms in the systems containing transition metals, for all ROHF (SAHF, CSF-ROHF, and HS-ROHF), ICE-CI, and CASSCF calculations. For the gadolinium dimer, scalar relativistic effects were considered by using X2C, together with the basis set X2C-TZVPall.^68^

## 3 Definition of Guess Starting Orbitals

The methodology described in the theory section above allows for determining the ROHF vector-coupling coefficients on an arbitrary number of open-shells that may consist of a given CSF. A second crucial step is to determine which type of starting molecular orbital may be part of an open-shell. This, in principle, will always involve some freedom of choice from the user’s perspective. Nevertheless, we draw some general guidelines with the aim to rendering the entire methodology as closely to the black box as possible.

In our experience, the usage of the atomic valence active space (AVAS)^69^ procedure constitutes a systematic way to generate metal centered initial guess orbitals suitable for transition metal dimers, trimers, etc. These orbitals are then used as a guess for a SAHF calculation, where the open-shell is defined as the SOMOs of the system. Since the SAHF problem is already set for the average of spin-coupling situations of the target multiplicity, the resulting orbitals should be better suited as a guess for the ROHF calculation on a specific CSF. The converged SAHF orbitals are localized and used as a guess themselves for the CSF-ROHF. Care must be exercised in order for the localized orbitals to reflect the chemical situation of interest, and we point out that, although recommended, not every chemical problem requires the localization step, as can be seen on the [Co(^1^L_N_)_2_] system presented in this study.

One can also recanonicalize the localized orbitals before using them as a guess for the following CSF-ROHF calculation. In ORCA, this is achieved by using the standalone program *orca_blockf*, *w*hich performs a block diagonalization of the Fock matrix for a given subspace.

**Figure 2:**
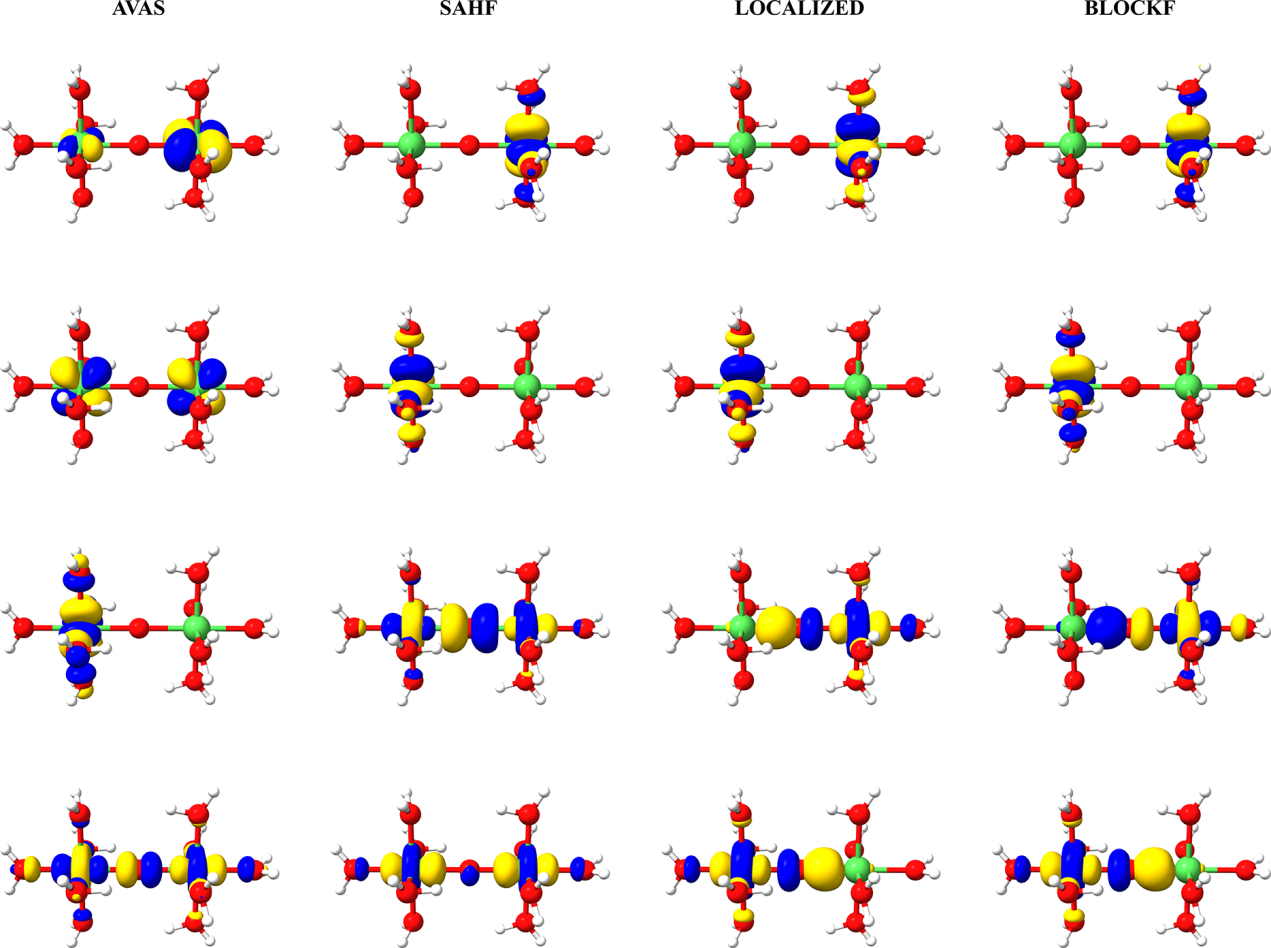
Example sequence of molecular orbital transformations used in the protocol for spin-couplings between metal centers. From left to right: the AVAS orbitals; the SAHF orbitals obtained using the AVAS guess; the localized orbitals obtained from the canonical SAHF ones; the recanonicalized orbitals obtained by using the orca_blockf utility; and the CSF-ROHF-optimized orbitals obtained from the localized and recanonicalized SAHF orbitals.

An example of the protocol used for the setup of the guess orbitals for the CSF-ROHF method is shown in Figure 2 for the [Ni_2_O(H_2_O)_10_]^2+^ system, where we want to set the ROHF problem for two open-shells, each with the singly occupied e_g_ orbitals from a distinct nickel center.

First, a SAHF calculation for 1 open-shell with 4 orbitals and 4 electrons in an overall singlet state was performed using, as an initial guess, the AVAS orbitals. The SAHF converged orbitals were then localized and recanonicalized before being used as the initial guess for the CSF-ROHF calculation, where two open-shells were set containing the respective nickel orbitals. As seen in Figure 2, the CSF-ROHF orbitals differ in comparison to the localized SAHF orbitals, reflecting the relaxation of the former for the given CSF.

## 4 Scaling and Comparison with CASSCF Timings

**Figure 3:**
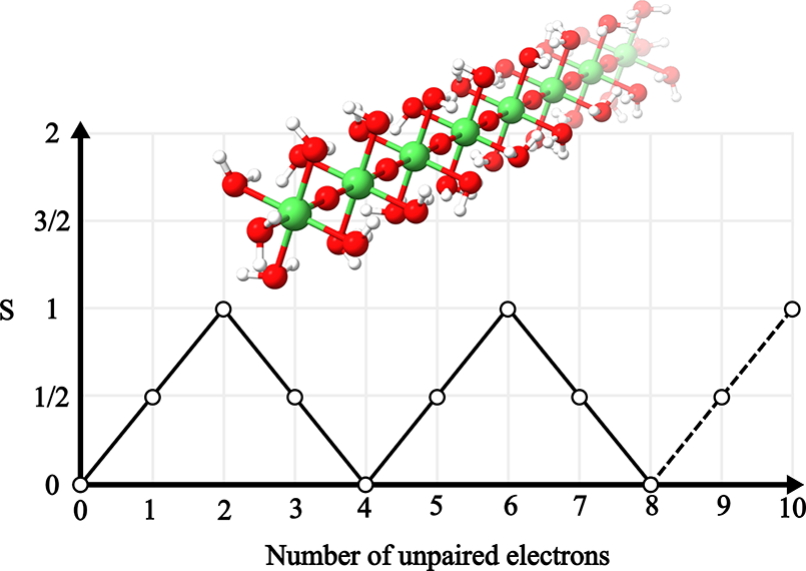
Model Ni chain used for timings and the branching diagram representation of the alternating spin-coupling between the Ni centers. Dashed line represents a continuation of the diagram as more Ni centers are added.

In the next step, we compare, in this section, the performance of the aforementioned CSF-ROHF procedure. For this purpose, we perform a series of calculations on [Ni(H_2_O)6]^2+^ as well as the [Ni(H_2_O)_5_]_*n*_O_*n*–1_, *n* = 2–10 chain, where the number of nickel atoms is gradually increased from 2 to 10 Ni atoms (from 303 to 2535 basis functions). Each nickel added is coupled antiferromagnetically with the previous one, resulting in a series of CSFs following the spin-coupling scheme, as shown in Figure 3.

Following the process described in Section 3, for each chain size, guess orbitals were obtained by first converging a SAHF calculation where the open-shell consists of the degenerate set of e_g_ orbitals of the Ni centers to low multiplicity (*S* = 0 for even numbers of Ni centers and *S* = 1 for odd numbers). The converged orbitals were then localized using the Pipek–Mezey^70^ localization scheme, which were then used as initial guess orbitals for all CSF-ROHF and CASSCF calculations. The CSF-ROHF calculations were set up following the respective branching diagram CSF for the alternating spin-coupling (Figure 3). The CASSCF calculations were performed for the lowest root of a minimal active space consisting of the d orbitals of the nickel centers with appropriate multiplicity. The HS-ROHF calculations were set using quasi-restricted orbitals (QROs),^71^ obtained from a UHF calculation on the same multiplicity as an initial guess. The usage of QROs usually ensures quick convergence of the HS-ROHF.

**Figure 4:**
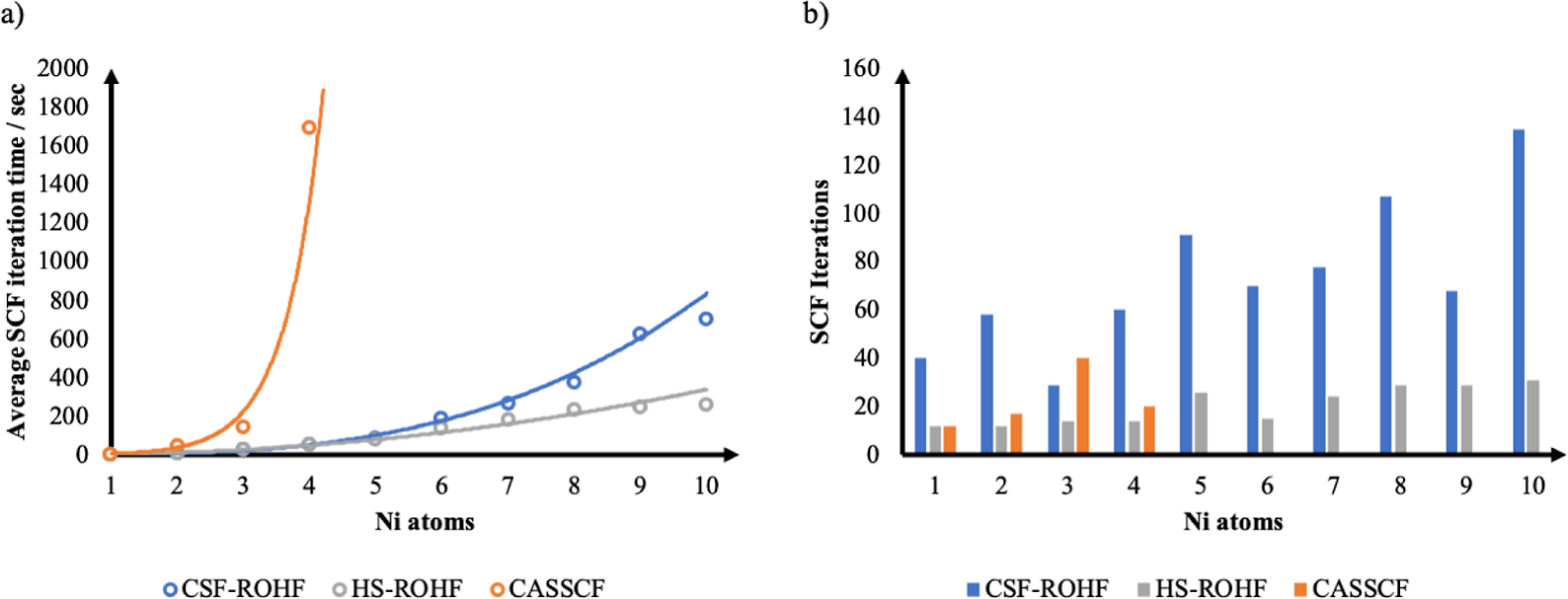
a) Average SCF iteration time for CSF-ROHF, HS-ROHF, and CASSCF calculations on the Ni chain with an increasing number of Ni atoms. (b) Number of SCF cycles needed to achieve convergence of the respective methods.

The computed average times per SCF iteration and total number of SCF iterations needed to achieve convergence are presented in Figure 4 as a function of the number of Ni centers in [Ni(H_2_O)6]^2+^ and [Ni(H_2_O)_5_]_*n*_O_*n*–1_, *n* = 2–10. All comparisons were performed in an Intel cluster using 8 cores and 4 GB of RAM per core.

As shown in Figure 4a, the CASSCF calculation presents the expected steep scaling with the increasing number of Ni(II) centers and becomes prohibitively expensive beyond 4 centers [corresponding to active spaces larger than (32,20)]. By contrast, HS-ROHF and CSF-ROHF scale proportionally to *N*_basis_^2^ if no precautions are taken for the calculation of the Coulomb term. We point out that in CSF-ROHF, a number of Fock matrices proportional to the number of open-shells needs to be processed, while in HS-ROHF, there are always only 2 Fock matrices in the procedure. The presented scaling for the CSF-ROHF method allows its application on problems where CASCF calculations are unfeasible.

It is also worth mentioning the number of SCF iterations needed to achieve convergence. Overall, the CSF-ROHF calculations converge in a reasonable number of iterations, ranging between 40 and 135 iterations, showing that, at least for the systems studied in this work, a rather smooth convergence behavior was observed. While this convergence behavior is, of course, closely related to the specific algorithm used to solve the SCF equations, these results at least indicate that the CSF-ROHF method is not expected to create any convergence issues that are worse than those of the parent HS-ROHF method.

## 5 Validation

**Figure 5:**
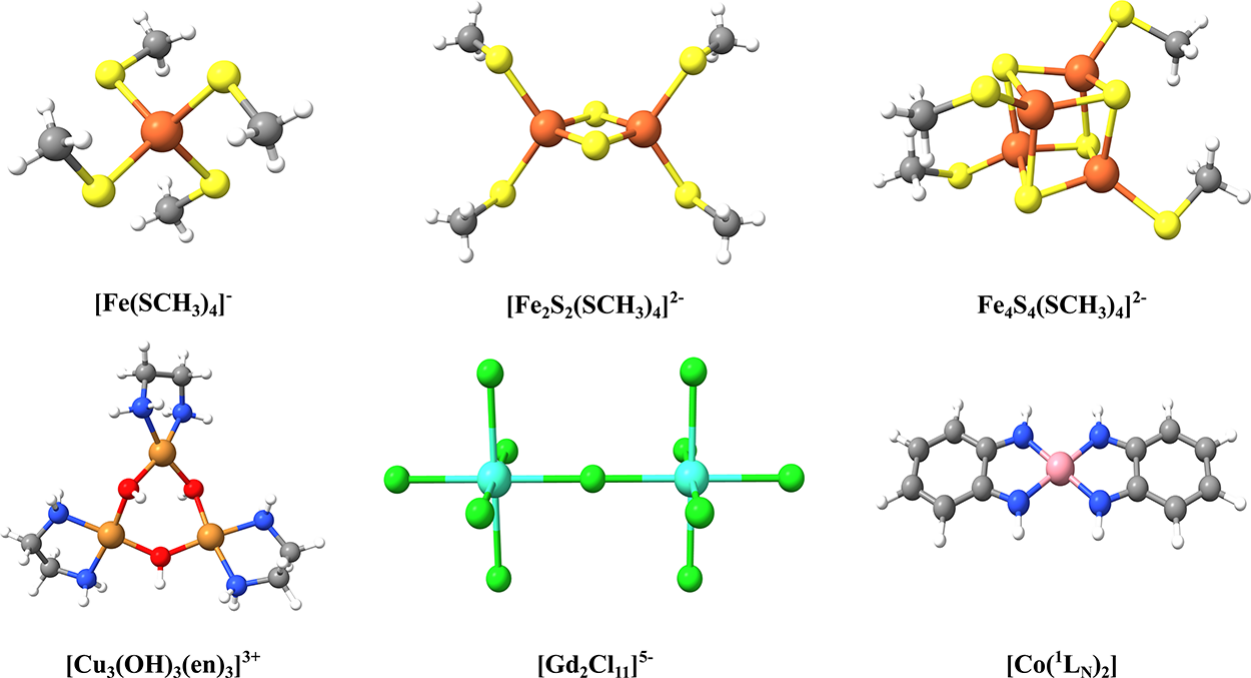
Structures of all systems studied in the present paper.

The validation of the presented CSF-ROHF methodology was performed in a study set that consisted of a series of monometallic and polymetallic complexes belonging to the transition metal and lanthanide families (Figure 5). In particular, the monomer [Fe(SCH_3_)_4_]^−^ was used to probe a series of ROHF-SCF solutions of Hund and non-Hund states, while the dimer complexes [Fe_2_S_2_(SCH_3_)_4_]^2–^ and [Gd_2_Cl_11_]^5–^ were used to probe the ROHF solutions of ferromagnetic and antiferromagnetic states. The trimer [Cu_3_(OH)_3_(en)_3_]^3+^, the tetramer [Fe_4_S_4_(SCH_3_)_4_]^2–^, and the monomer [Co(^1^L_N_)_2_], ^1^L_N_ = C_6_H_4_(NH_2_) were used to emphasize the generality of the presented CSF-ROHF methodology, by investigating more complex spin-coupling situations, which involve ligand orbitals in metal–ligand covalency or metal–ligand radical interactions.

### 5.1 Probing Hund and Non-Hund States

As a first application of the presented CSF-based ROHF, we turn to the [Fe(SCH_3_)_4_]^−^ complex, which contains an Fe(III) center in an S_4_ symmetry coordination environment, resulting in a ground state electronic configuration with , following Hund’s rule, where each of the 3d orbital of the iron center is singly occupied. The CSF representing this spin-coupling situation is [+ + + + +], and its branching diagram representation can be seen in Figure S1.

Keeping the occupation number of the five 3d orbitals, it is also possible to construct CSFs for the non-Hund states with  and . Maintaining the MO energy ordering of the  state, 5 electrons in 5 orbitals lead to 4 CSFs coupled to  and 5 CSFs coupled to , representing the aforementioned non-Hund states of [Fe(SCH_3_)_4_]^−^. Each of these CSFs has a unique branching diagram, which allows for the determination of the *b*^*IJ*^ vector-coupling coefficients for each case, as shown in Table 2.

**Table 2:** Number of Open-Shells and Determined Values for *b*^*IJ*^

multiplicity	CSF	number of open-shells	*b*^*IJ*^
6	+ + + + +	1	2
4	+ + + + −	2	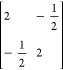
	+ + + – +	3	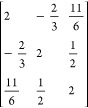
	+ + – + +	3	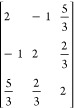
	+ – + + +	3	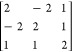
2	+ + + – −	2	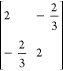
	+ + – – +	3	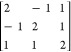
	+ – + + −	4	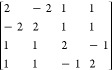
	+ + – + −	4	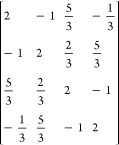
	+ – + – +	5	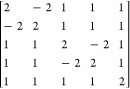

The number of open-shells for each CSF is defined by the number of sets of parallel spin-coupling orbitals. As an example, the CSF [+ + – + +] has three groups of parallel spins: the first two +, the following -, and the last two + +, leading to a ROHF problem with 3 open-shells. Table 2 also shows the respective numbers of open-shells for each CSF studied.

With the values of the ROHF vector-coupling coefficients determined, the calculation of the total electronic energy for each of the CSF-ROHF cases can be carried out. The total energies are presented in Table 3, as well as the relative energy with respect to the converged HS state.

**Table 3:** ROHF Energies for the Different Multiplicities and CSFs Arising from 5 Singly Occupied Orbitals in [Fe(SCH_3_)_4_]^−^

multiplicity	CSF	*E*/hartree	Δ*E*/hartree	Δ*E*/cm–1
6	+ + + + +	–3011.14,803	0	0
4	+ + + – +	–3011.04201	0.10601	23,267
	+ + + + –	–3011.03221	0.11582	25,419
	+ + – + +	–3011.03108	0.11695	25,667
	+ – + + +	–3010.98121	0.16682	36,612
2	+ + + – –	–3010.93950	0.20852	45,766
	+ + – + –	–3010.92805	0.21998	48,279
	+ + – – +	–3010.91783	0.23020	50,522
	+ – + – +	–3010.89386	0.25416	55,783
	+ – + + –	–3010.89167	0.25636	56,265

It should be emphasized that for the case of the unique sextet CSF, both CSF-ROHF and CASSCF (5,5) solve the same problem, since from this CSF within the defined active space, no excitations can be performed. Hence, the CASSCF wave function will also consist of only one sextet CSF and the orbital optimization step reduces to the ROHF problem. This is true for any HS situation where the active space is defined to include only the singly occupied orbitals.

### 5.2 Probing Ferromagnetic and Antiferromagnetic Couplings

We now turn to the case of the iron dimer [Fe_2_S_2_(SCH_3_)_2_]^2–^, which consists of two HS Fe(III) centers bridged by sulfides. Assuming two local  systems, we constructed the ROHF problem for the ferromagnetic coupling (*S* = 5) and antiferromagnetic coupling (*S* = 0) situations. For *S* = 5, only one CSF can be constructed, where the 10 unpaired electrons are all coupled parallel to each other. However, there are 42 CSFs that can be constructed for 10 electrons in 10 orbitals coupled to a singlet state; these can be referred to as the “neutral” CSFs since both Fe centers have the same charge. Among these 42, we selected the CSF with [+ + + + + – – – −] coupling as the illustrative example of the antiferromagnetic coupling between the two Fe centers.

The CSF-ROHF problem was then set up in the following way. For the HS *S* = 5 state, we have 1 open-shell with 10 orbitals and 10 electrons. For the LS *S* = 0 state, two open-shells are employed, each containing 5 electrons and the 3d orbitals of the Fe(III) centers. For comparison, CASSCF calculations were also performed on both spin states using the same set of guess orbitals. The energy differences between both spin states are shown in Table 4.

**Table 4:** Energy Difference between the *S* = 0 (LS) and *S* = 5 (HS) States Obtained from the CSF-ROHF, CAS-ICE (10,10) Using Both HS ROHF Orbitals (HS-ROHF) and LS ROHF Orbitals (LS-ROHF), and CASSCF (10,10) Calculations on the [Fe_2_S_2_(SCH_3_)_2_]^2–^, the *S* = 0 (LS) and *S* = 7 (HS) States Obtained from the CSF-ROHF, CAS-ICE (14,14) with CSF-ROHF Orbitals and CASSCF (14,14) Calculations on [Gd_2_Cl_11_]^5–^, and the *S* = 0 (LS) and *S* = 9 (HS) States Obtained from the CSF-ROHF, CAS-ICE (18,18) with CSF-ROHF Orbitals, and CASSCF (18,18) Calculations on [Fe_4_S_4_(SCH_3_)_4_]^2–^

	CSF-ROHF	CAS-ICE (HS-ROHF)	CAS-ICE (CSF-ROHF)	CASSCF
[Fe_2_S_2_(SCH_3_)_2_]^2–^				
Δ*E* (LS – HS)/cm^–1^	843.89	–1320.90	–1332.59	–1765.89
[Gd_2_Cl_11_]^5–^				
Δ*E* (LS – HS)/cm^–1^	–1.4089	0.8784	0.6936	0.7735
[Fe_4_S_4_(SCH_3_)_4_]^2–^				
Δ*E* (LS – HS)/cm^–1^	1740.65	–2045.87	–2245.77	–2754.76

**Figure 6:**
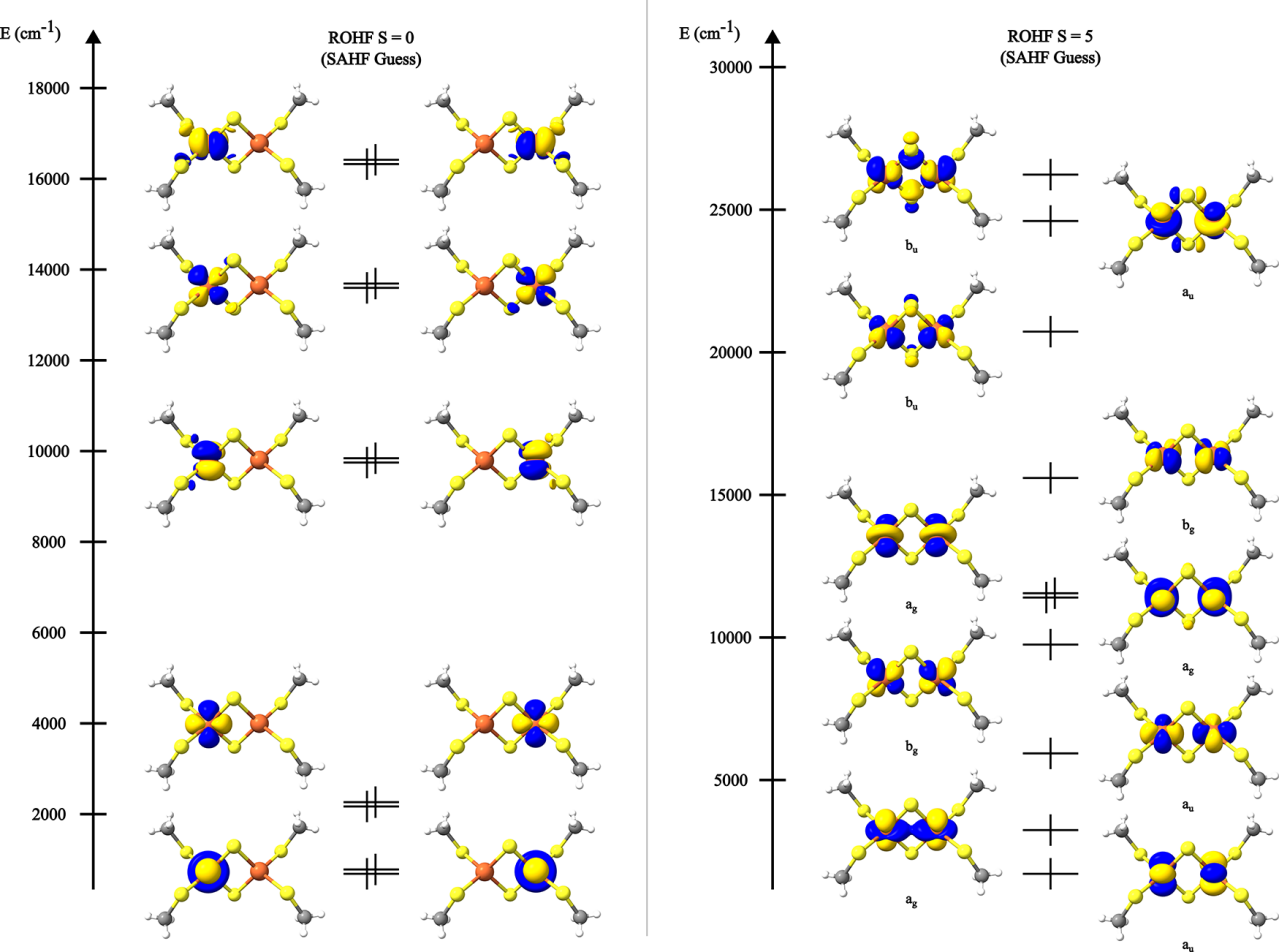
ROHF canonical molecular orbitals of [Fe_2_S_2_(SCH_3_)_4_]^2–^ optimized for the CSF [+ + + + + – – – – −], *S* = 0 (left) and the CSF [+ + + + + + + + + +], *S* = 5 (right).

Inspection of Table 4 shows that, in a first approximation, there is a disagreement between the CSF-ROHF and CASSCF energy differences between the two spin states, with the CSF-ROHF energy difference favoring the *S* = 5 state. The reasoning behind this discrepancy follows from the mechanism of antiferromagnetic coupling between two metal centers. As discussed in more detail elsewhere,^56,72−74^ in order to properly describe the coupling between the two Fe centers, not only the “neutral” CSFs are needed but also the “ionic” CSFs, in which one electron is transferred from one iron center to the other. Since the ROHF calculation was performed exclusively for one of the “neutral” CSFs, it fails to capture the antiferromagnetic coupling in its entirety. Hence, in a subsequent step, the missing “ionic” CSFs can be included on the basis of CAS-ICE calculations with the ROHF-optimized orbitals (Figure 6). This lowers the energy of the *S* = 0 state by 2084 cm^–1^, due to mixing with the “ionic” CSFs, providing consistent energy splittings between CASSCF and CSF-ROHF/CAS-ICE. We emphasize that even though there is an energy lowering resulting from the mixing of other CSFs, the wave function resultant from the CSF-ROHF/CAS-ICE calculation is still dominated by the [+ + + + + – – – – −] CSF (∼98%, Table 5). For comparison, CAS-ICE calculations were also performed using the HS ROHF solution, where the obtained orbitals were just localized before the CI. In this case, the resulting wave function is no longer dominated by the [+ + + + + – – – – −] CSF, showing the inadequacy of the HS solution as a basis for subsequent correlated calculations of the lower multiplicities (consistent with ref (35)).

**Table 5:** CSFs Weights for the Wavefunctions of the Dimers [Fe_2_S_2_(SCH_3_)_2_]^2–^ and [Gd_2_Cl_11_]^5–^

	CAS-ICE (HS-ROHF)	CAS-ICE (LS-ROHF)	CASSCF
[Fe_2_S_2_(SCH_3_)_2_]^2–^			
*S* = 0	0.37 [+ – + + + – – – + −]	0.98 [+ + + + + – – – – −]	0.96 [+ + + + + – – – – −]
	0.12 [++ – + + – – – + −]		
	0.12 [+– + + + – – + – −]		
*S* = 5	1.0 [+ + + + + + + + + +]	1.0 [+ + + + + + + + + +]	1.0 [+ + + + + + + + + +]
[Gd_2_Cl_11_]^5–^			
*S* = 0	1.0 [+ + + + + + +– – – – – – −]	1.0 [+ + + + + + + – – – – – – −]	1.0 [+ + + + + + + – – – – – – −]
*S* = 7	1.0 [+ + + + + + + + + + + + + +]	1.0 [+ + + + + + + + + + + + + +]	1.0 [+ + + + + + + + + + + + + +]
[Fe_4_S_4_(SCH_3_)_4_]^2–^			
*S* = 0	0.97 [+ + + + + + + + + – – – – – – – – −]	0.97 [+ + + + + + + + + – – – – – – – – −]	0.97 [+ + + + + + + + + – – – – – – – – −]
*S* = 9	1.0 [+ + + + + + + + + + + + + + + + + +]	1.0 [+ + + + + + + + + + + + + + + + + +]	1.0 [+ + + + + + + + + + + + + + + + + +]

Likewise to the monomer case, the mixing of CSFs discussed above is not present in the HS state, which is uniquely defined by a single CSF. This renders the CASSCF calculation to be exactly the ROHF problem, leading to the same final electronic energy obtained by both methods (Table S1).

The same procedure used for the iron dimer is not restricted only to transition metals, but it is generally applicable to any situation where the interacting open-shells can be defined. As an example, we used a model Gd(III) dimer [Gd_2_Cl_11_]^5–^ where the gadolinium centers have f^7^ electronic configuration in a local  spin state. Once again, by employing CSF-ROHF, CASSCF, and CAS-ICE calculations, both ferromagnetic (*S* = 7) and antiferromagnetic (*S* = 0) coupling situations may be probed (Table 4). In contrast to [Fe_2_S_2_(SCH_3_)_2_]^2–^, in the case of [Gd_2_Cl_11_]^5–^, the energy difference between the ferromagnetic and antiferromagnetic coupling situations is negligible with all methods applied. This is due to the lack of covalency between metal and ligands, causing the two gadolinium centers to be essentially isolated from each other, as is also reflected in the ROHF canonical MOs obtained for both spin-coupling situations (see Supporting Information).

Going up in complexity, we address the model iron–sulfur cluster [Fe_4_S_4_(SCH_3_)_4_]^2–^ (Figure 5). It is generally accepted^57^ that the spin situation in this cluster involves mixed valence Fe(II)–Fe(III) pairs, also referred as Fe^2.5+^–Fe^2.5+^, that couple ferromagnetically within themselves and antiferromagnetically with the pair on the opposite face of the cube, resulting in an overall singlet state.

**Figure 7:**
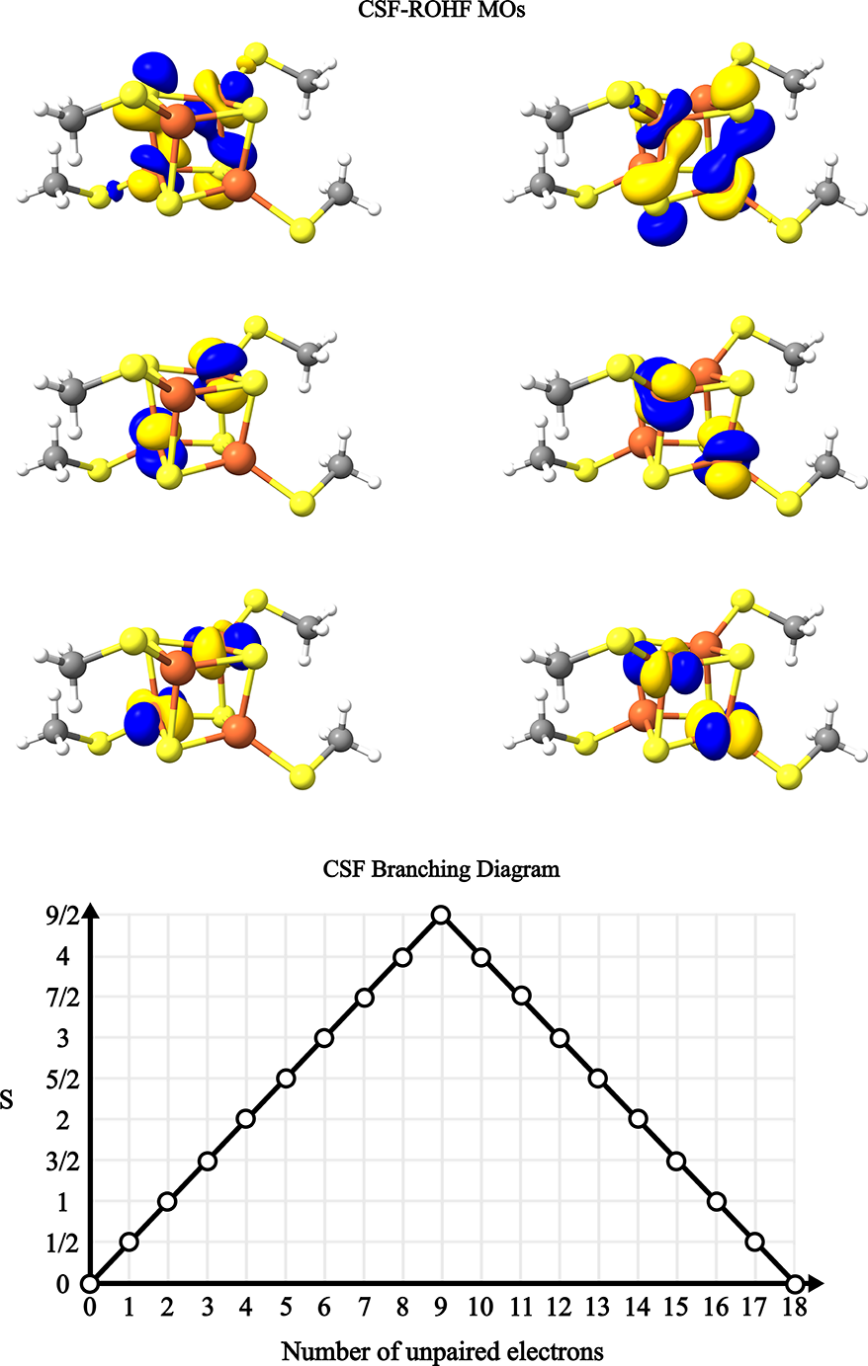
Representative open-shell molecular orbitals (top) obtained for the [Fe_4_S_4_(SCH_3_)_4_]^2–^ cluster using the 2 open-shell CSF coupling scheme shown in the branching diagram (bottom).

With the accepted spin situation in mind, the open-shells of the system are to be specified using a set of 18 orbitals and 18 electrons resulting from 2 Fe(II) and 2 Fe(III) atoms. This gives rise to the question of how to define the open-shells for this system. Although we can use the CSF represented on the branching diagram of Figure 7, there is still ambiguity on which orbitals to include on the two open-shells. Since there are 4 iron centers that can be paired and the optimized structure of this model is not a perfect cube, there are 3 distinct possible iron pairings to consider.

Following the procedure described in Section 3, by first performing a SAHF calculation with just one open-shell containing all iron atoms and an overall singlet state, we obtain converged orbitals that, after localization with the Pipek–Mezey scheme,^70^ are already paired in a distinct situation. These orbitals can be used as the guess orbitals on the CSF-ROHF problem for *S* = 0 CSF, where the two open-shells are defined according to the pairing of SAHF orbitals. For comparison, we also performed the conventional HS ROHF for the *S* = 9 state. The ROHF energy differences between the two spin-coupling situations are listed in Table 4.

Again, the correct description of the antiferromagnetic coupling can be obtained only after the inclusion of the missing “ionic” CSFs in a subsequent CI calculation for the *S* = 0 problem. In line with the [Fe_2_S_2_(SCH_3_)_2_]^2–^ dimer results, after CI, the wave function is still dominated by the CSF [+ + + + + + + + + – – – – – – – – −] (Table 5), this time for both CI calculation employing MOs obtained from the two spin-coupling ROHF problems.

As has been already shown,^35,57^ probing the actual magnetic structure of the ground state of these systems requires expansion of the active space to include all relevant metal and ligand-based orbitals participating in metal–ligand covalent interactions. In the context of approximate CI, further CSF mixing with the “neutral” CSF will be introduced, which, in principle, would require further orbital relaxation than the CSF-ROHF provides before robust results could be achieved. This is beyond the scope of the current work.

### 5.3 Probing Complex Spin-Coupling Situations

In the previous section, we encountered the problem of defining the open-shells in spin-coupling situations where this choice was somewhat straightforward. However, this is not always the case, particularly in multimetallic systems with more than two metal centers. A characteristic case refers to the copper trimer [Cu_3_(OH)_3_(en)_3_]^3+^. In this system, there are 3 Cu(II) centers each with local . In this scenario, the three centers can couple to an overall quartet state or to a doublet.

For the quartet state, there is only one possible CSF that can be constructed, where all unpaired spins are coupled parallel to each other. In fact, for this case, the open-shell definition is unique since there is only one open-shell with 3 electrons and 3 orbitals.

In contrast, for the doublet state, two possible CSFs can be constructed. One of them, the one with spin-coupling [+ – +], presents no further ambiguities since the three open-shells can be attributed to each Cu center. Now for the [+ + −] CSF, the question arises of which Cu orbitals should consist of each of the two open-shells. Indeed, we can set three CSF-ROHF problems for this spin-coupling situation, where we include different pairs of Cu centers that may be included in the first open-shell.

**Figure 8:**
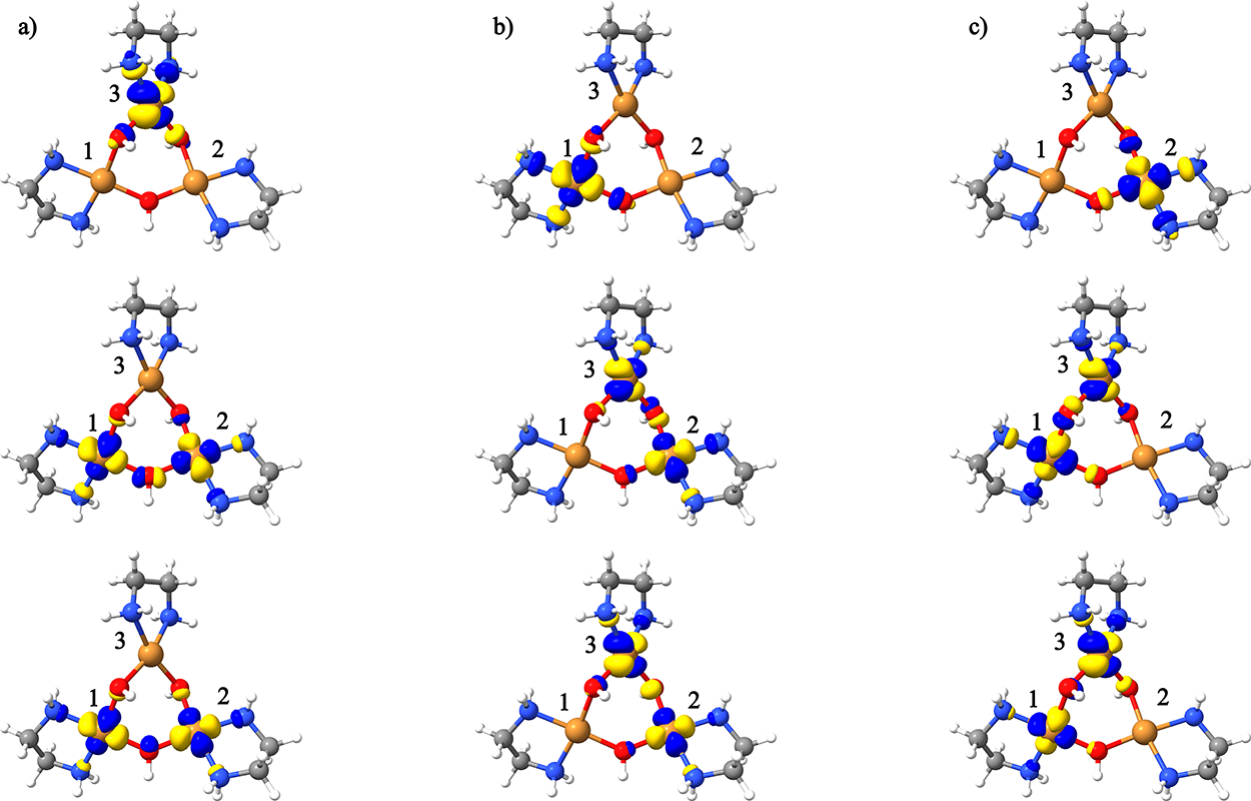
Open-shell molecular orbitals for the CSF [+ + −] by setting the open-shells as (a) (Cu1,Cu2)(Cu3), (b) (Cu2,Cu3)(Cu1), and (c) (Cu1,Cu3)(Cu2).

As can be seen in Table 6, the three possibilities result in similar single point energies, in accordance with the local ∼ *C*_3_ symmetry of the Cu centers of the complex (Figure 8), which makes the possible [+ + −] CSFs as well as the [+ – +] CSF essentially identical. Nevertheless, this serves to show that the determination of the vector-coupling coefficients defines only the spin-coupling situation, and the guess orbitals included in the open-shells still need to be chosen prior to the ROHF calculation.

**Table 6:** Calculated Energies for Each CSF of Complex [Cu_3_(OH)_3_(en)_3_]^3+^

CSF	[+ + +]	[+ – +]	[+ + −] 1	[+ + −] 2	[+ + −] 3
energy/*E*_h_	–5710.82308	–5710.82291	–5710.82293	–5710.82291	–5710.82292
Δ*E*/cm^–1^	0	34.4200	37.2519	36.2623	33.8176

### 5.4 Probing Metal–Ligand Radical Spin-Couplings

As a last case, we expand our discussion to include spin-coupling situations between organic radicals and transition metal centers. A characteristic example refers to the complex [Co(^1^L_N_)_2_], ^1^L_N_ = C_6_H_4_(NH_2_), which falls into a wide category of coordination compounds of transition metals bearing “non-innocent” ligands. The accepted ground state electronic structure for this system consists of three open-shell orbitals coupled to a total spin .^75^

**Figure 9:**
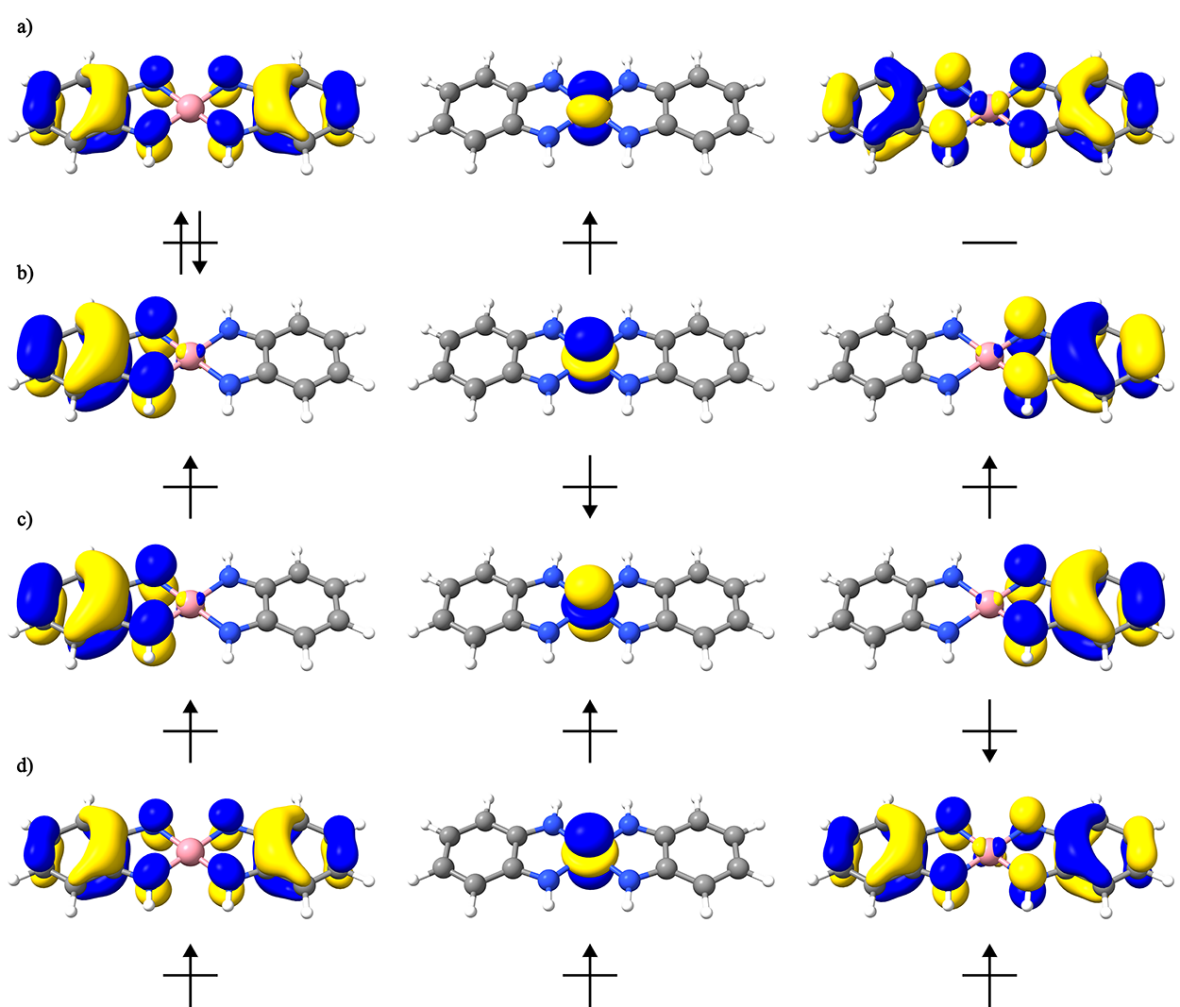
Optimized open-shell MOs for the four different CSFs for the complex [Co(^1^L_N_)_2_] (a) closed-shell doublet, (b) [+ + −] doublet CSF, (c) [+ – +] doublet CSF, and (d) [+ + +] quartet CSF.

Keeping this structure in mind, four spin-coupling situations were defined, giving rise to four possible CSFs. A doublet CSF where there is only one open-shell, the two possible CSFs for three electrons coupling to a doublet state, [+ + −] and [+ – +] (center and right branching diagrams of Figure 1), and the quartet CSF [+ + +]. The CSF-ROHF calculations for the [+ + −], [+ – +], and [+ + +] CSFs were performed using the initial guess orbitals of the optimized MOs obtained from the ROHF on the one open-shell doublet CSF (Figure 9a), with no further localization procedure employed.

The CSF-ROHF optimized orbitals are shown in Figure 9, and the calculated ROHF energies are presented in Table 7. One can readily see that all ROHF solutions for the CSFs with 3 orbitals and 3 electrons are lower in energy than the closed-shell doublet. We also performed CAS-ICE calculations using different ROHF solutions in order to obtain a more correct description of the spin-coupling situation of the system.

**Table 7:** Calculated Energies for the Three CSFs of Complex [Co(^1^L_N_)_2_] and the Energy Difference in Respect to the One Open-Shell Doublet State

CSF	[+]	[+ + −]	[+ – +]	[+ + +]
ROHF				
energy/*E*_h_	–2060.72627	–2060.77886	–2060.77817	–2060.78086
Δ*E*/cm^–1^	0	–11,540.8	–11,390.7	–11,980.3
CAS-ICE				
energy/*E*_h_	–2060.76480	–2060.78472	–2060.78462	–2060.78086
Δ*E*/cm^–1^	0	–4371.9	–4349.4	–3524.9

As can be seen in Table 8, after the CI calculation using either the ROHF orbitals obtained for the [+ – +] or [+ + −] CSFs, the wave function is over 70% composed of the [+ + −] one. This, together with the energies ca. 847 cm^–1^ lower than the quartet state reflects the diradical character of the ligands, in accordance with the accepted electronic structure.^75^ The difference in the percentage composition for these two CI calculations reflects the two CSF-ROHF solutions for the [+ – +] and [+ + −] CSFs.

**Table 8:** CAS-ICE Wavefunction Composition Using Different CSF-ROHF Orbitals (Figure 9) in Terms of the Possible CSFs for 3 Electrons in 3 Orbitals^a^

mult	ROHF CSF	[2 + 0] (%)	[0 + 2] (%)	[+ + −] (%)	[+ – +] (%)	[+ + +] (%)
2	[+]	76	24	0	0	0
	[+ – +]	1	2	70	27	0
	[+ + −]	2	1	75	22	0
4	[+ + +]	0	0	0	0	100

a A number 2 represents a doubly occupied orbital and a number 0 represents a virtual orbital.

## 6 Conclusions

In this study, we presented a method to calculate the ROHF vector-coupling coefficients for a given CSF, which allows for the calculation of proper spin-eigenfunctions of arbitrary spin-coupling situations. The method is generally applicable to systems where one defines an open-shell consisting of only singly occupied orbitals, meaning that the occupation number *n*^μ^ for the open-shell part of the restricted open-shell Fock operator is always 1. This allows the formulation of a general spin ROHF methodology starting from a given CSF that can be used to provide a SCF solution for a given spin-coupling situation.

CSF-ROHF makes use of an efficient infrastructure to compute the vector-coupling vectors, by computing the needed two-body ⟨Φ|*E*_*u*_^*t*^*E*_*t*_^*u*^|Φ⟩ matrix elements directly in the CSF basis of the problem, with no expansion in Slater determinants required. It scales close to quadratically with system size and shows satisfactory convergence performance.

The method is validated to probe the Hund and non-Hund CSFs of the complex [Fe(SCH_3_)_4_]^−^, the ferromagnetic and antiferromagnetic spin-coupling situations of the [Fe_2_S_2_(SCH_3_)_4_]^2–^ and [Gd_2_Cl_11_]^5–^ dimers, and the spin-coupling situations on the trimer [Cu_3_(OH)_3_(en)_3_]^3+^, the tetramer [Fe_4_S_4_(SCH_3_)_4_]^2–^, and the complex [Co(^1^L_N_)_2_], ^1^L_N_ = C_6_H_4_(NH_2_). Hence, classes of antiferromagnetically coupled systems ranging from multimetallic chains, metal clusters, and extended metal–ligand radicals can be treated.

Ongoing efforts are directed toward the development of dynamic correlation methods that start from the CSF-ROHF reference states. Next to many-body perturbation and coupled cluster approaches, obvious choices are methods related to approximate full-CI schemes such as DMRG,^32^ FCI-QMC,^33−35^ or the CIPSI^36,37^/ICE^38,39^ family of selecting CI methods. We foresee exciting applications of such methods to challenging chemical problems in (bio)chemistry, catalysis, and material sciences.

## Supporting Information Available

Branching diagrams for the sextet, quartet, and doublet multiplicities of [Fe(SCH_3_)_4_]; CSF-ROHF molecular orbitals for the CSF [+ + + + + + + – – – – – – −] of [Gd_2_Cl_11_]^5–^; and HS-ROHF molecular orbitals for [Gd_2_Cl_11_]^5–^ (PDF)
