# Supplementary material for: Restricted Open-Shell Hartree–Fock Method for a General Configuration State Function Featuring Arbitrarily Complex Spin-Couplings
Source: J Phys Chem A. 2024 Jun 17;128(25):5041–53. doi: 10.1021/acs.jpca.4c00688 (PMC11215774; doi:10.1021/acs.jpca.4c00688)
Supplement: Supplementary file 1 — jp4c00688_si_001.pdf [file jp4c00688_si_001.pdf]

Supporting Information

A Restricted Open-Shell Hartree-Fock Method for a General Configuration State Function Featuring Arbitrarily Complex Spin-Couplings

Tiago Leyser da Costa Gouveia[1], Dimitrios Maganas[1] and Frank Neese[1*]

Table of Contents

1. Ni chain........ S3

A. Geometries........ S3

2. $[Fe(SCH_3)_4]^-$........ S20

A. Geometry........ S20

B. Branching diagrams........ S21

C. Sample Inputs........ S21

3. $[Fe_2S_2(SCH_3)_4]^{2-}$........ S24

A. Geometry........ S24

B. Sample Inputs........ S24

4. $[Gd_2Cl_{11}]^{5-}$........ S27

A. Geometry........ S27

B. Molecular Orbitals........ S27

5. $[Fe_4S_4(SCH_3)_4]^{2-}$........ S29

A. Geometry........ S29

6. $[Cu_3(OH)_3(en)_3]^{3+}$........ S30

A. Geometry........ S30

7. $[Co(^1L_N)_2]$........ S31

A. Geometry........ S31

1. Ni chain

A. Geometries

i. 1 Ni:

```
Ni  0.00000000000000      0.00000000000000      0.00000000000000
O   2.08738999752000      0.00000000000000      0.00000000000000
O  -2.08738999752000      0.00000004408251      0.00000000000000
O  -0.00000000145727      2.08738999752000      0.00000000000000
O   0.00000000072864     -2.08738999752000     -0.00000000000000
O  -0.00000000072864      0.00000000072864     -2.08738999752000
O   0.00000000072864      0.00000000072864      2.08738999752000
H   2.65763473155719      0.78999235683467     -0.00000131854335
H   2.65763485984455     -0.78999226522986     -0.00000000000000
H   0.00000000076434      2.65763446560963     -0.78999254420468
H   0.00000081701211      2.65763458772359      0.78999243939734
H  -0.78999215339847      0.00000066219845      2.65763502439174
H   0.78999239892974      0.00000000092769      2.65763469199836
H  -2.65763480169159      0.78999234525531      0.00000098542597
H  -2.65763477981872     -0.78999227327541     -0.00000000000000
H  -0.00000015005059     -2.65763446565846      0.78999254456285
H  -0.00000085447791     -2.65763459603914     -0.78999243697144
H  -0.78999241156412     -0.00000009255474     -2.65763467469341
H   0.78999214302993      0.00000000092769     -2.65763503766959
```

ii. 2 Ni:

```
Ni  0.00023500000000     -0.03373900000000      0.01222700000000
O   2.08762500000000     -0.03373900000000      0.01238000000000
O  -2.08715500000000     -0.03373900000000      0.01207400000000
O   0.00023400000000      2.05353900000000      0.03382800000000
O   0.00023700000000     -2.12101700000000     -0.00937400000000
O   0.00038800000000     -0.01213900000000     -2.07505100000000
O   0.00008300000000     -0.05534000000000      2.09950500000000
H   2.61058100000000      0.71468400000000     -0.32528900000000
H   2.56534500000000     -0.85014500000000     -0.21914900000000
H  -0.00163000000000      2.88884100000000     -0.46497400000000
H  -0.00089600000000      2.23334200000000      0.99682200000000
H  -2.61018800000000      0.71460900000000     -0.32563500000000
H  -2.56494200000000     -0.85019200000000     -0.21913500000000
H  -0.00038700000000     -2.40844200000000      0.92301900000000
H  -0.00104300000000     -2.88919300000000     -0.60427300000000
H  -0.77788800000000      0.16732800000000     -2.63125000000000
H   0.77877800000000      0.16729300000000     -2.63110300000000
Ni -0.00007000000000     -0.07694100000000      4.18678400000000
O  -0.00007100000000      2.01033700000000      4.20838500000000
O  -0.00006900000000     -2.16421900000000      4.16518300000000
O  -0.00022200000000     -0.09854200000000      6.27406200000000
O   2.08732000000000     -0.07694100000000      4.18693600000000
O  -2.08746000000000     -0.07694100000000      4.18663100000000
H   0.77786000000000      2.56448000000000      4.39857400000000
H  -0.77701800000000      2.56455500000000      4.40237500000000
H  -0.78503000000000     -2.62371100000000      4.51544300000000
H   0.78502100000000     -2.62366600000000      4.51521700000000
H   0.00089400000000      0.65369200000000      6.89081700000000
H  -0.00039400000000     -0.90243900000000      6.82099200000000
H   2.69821900000000      0.57470400000000      4.57292600000000
H   2.09496900000000      0.02452600000000      3.20133300000000
H  -2.69838200000000      0.57470800000000      4.57257500000000
H  -2.09494600000000      0.02444600000000      3.20101500000000
```

iii. 3 Ni:

```
Ni -0.02982700000000     -0.01276000000000     -0.00029800000000
O   2.05735600000000     -0.01482900000000     -0.02964200000000
O  -2.11701000000000     -0.01069000000000      0.02904700000000
O  -0.02764500000000      2.07461400000000      0.00772500000000
O  -0.03200900000000     -2.10013300000000     -0.00832000000000
O  -0.05916300000000     -0.00470700000000     -2.08746600000000
O  -0.00049100000000     -0.02081200000000      2.08687100000000
H   2.58156100000000      0.76700300000000     -0.28339700000000
H   1.95713400000000      0.01326100000000      0.96063300000000
H   0.42762100000000      2.65403800000000     -0.63063500000000
H   0.32787200000000      2.24275300000000      0.90379900000000
H  -2.45395000000000      0.89258600000000     -0.12579300000000
H  -2.05016500000000     -0.10005400000000      1.01314300000000
H  -0.02157400000000     -2.12294300000000      0.97976200000000
H   0.81299100000000     -2.50513000000000     -0.28193300000000
H  -0.85823200000000     -0.42713300000000     -2.45500200000000
H   0.69116200000000     -0.40004200000000     -2.56706100000000
Ni  0.02884500000000     -0.02886500000000      4.17403900000000
O   0.03102800000000      2.05850900000000      4.18206200000000
O   0.02666300000000     -2.11623800000000      4.16601600000000
O   0.05818200000000     -0.03691800000000      6.26120700000000
O   2.11602800000000     -0.03093400000000      4.14469500000000
O  -2.05833700000000     -0.02679500000000      4.20338300000000
H  -0.68230600000000      2.53857200000000      4.63874800000000
H  -0.19549700000000      2.02716300000000      3.21930200000000
H  -0.89097700000000     -2.39596700000000      3.98828400000000
H   0.11777600000000     -2.09627500000000      5.14794900000000
H   2.40054300000000      0.88319500000000      4.33265500000000
H   2.07654200000000     -0.10517600000000      3.16187700000000
H  -2.00636500000000      0.17844500000000      5.17019600000000
H  -2.54573200000000      0.69777300000000      3.77292500000000
Ni  0.08751800000000     -0.04497000000000      8.34837600000000
O   0.08970000000000      2.04240300000000      8.35639800000000
O   0.08533500000000     -2.13234400000000      8.34035300000000
O  -1.99966500000000     -0.04290100000000      8.37772000000000
O   2.17470000000000     -0.04704000000000      8.31903200000000
O   0.11685300000000     -0.05302300000000     10.43554400000000
H   0.55164200000000      0.73567800000000     10.81096300000000
H   0.51387900000000     -0.81514800000000     10.89472800000000
H  -0.80858600000000      2.38131100000000      8.53407700000000
H   0.16097700000000      1.99316500000000      7.36982800000000
H  -0.69708400000000     -2.65468000000000      8.59617100000000
H   0.04249700000000     -2.01304500000000      7.35259000000000
H  -2.19089500000000     -0.38382700000000      7.48086900000000
H  -2.57156900000000     -0.49953600000000      9.02187800000000
H   2.58306100000000     -0.89721200000000      8.57071000000000
H   2.17961100000000     -0.04034000000000      7.33064900000000
```

iv. 4 Ni:

```
Ni  0.03694200000000      0.09357200000000      0.00222500000000
O   2.12135500000000     -0.01784400000000      0.00491200000000
O  -2.04747100000000      0.20498800000000     -0.00046200000000
O   0.14831400000000      2.17781900000000      0.02883600000000
O  -0.07443100000000     -1.99067500000000     -0.02438600000000
O   0.04104500000000      0.12000200000000     -2.08499300000000
O   0.03283800000000      0.06714200000000      2.08944400000000
H   2.06613900000000      0.02448100000000      0.99628500000000
H   2.59947300000000     -0.83679000000000     -0.21611600000000
H  -0.71274800000000      2.60022400000000     -0.15529600000000
H   0.18084200000000      2.07133100000000      1.01667500000000
```

```
H  -1.93148200000000      0.20515300000000      0.99253600000000
H  -2.45300400000000     -0.65762100000000     -0.21171400000000
H   0.13403100000000     -2.01507800000000      0.94107000000000
H   0.56291700000000     -2.55589600000000     -0.49637600000000
H  -0.79411700000000      0.33133800000000     -2.53968200000000
H   0.70572600000000      0.73335500000000     -2.44979100000000
Ni  0.02873500000000      0.04071200000000      4.17666200000000
O   0.14010700000000      2.12495900000000      4.20327400000000
O  -0.08263700000000     -2.04353500000000      4.15005100000000
O   0.02463200000000      0.01428200000000      6.26388100000000
O   2.11314800000000     -0.07070300000000      4.17934900000000
O  -2.05567800000000      0.15212800000000      4.17397500000000
H   1.08187800000000      2.34074500000000      4.34112800000000
H   0.05055700000000      2.03724900000000      3.21956000000000
H  -0.84478000000000     -2.42371700000000      3.67878900000000
H  -0.30075900000000     -2.03755900000000      5.11440900000000
H   2.00987000000000     -0.26205800000000      5.14841500000000
H   2.30538800000000     -0.93026400000000      3.76085000000000
H  -2.02164000000000      0.14815200000000      5.16421000000000
H  -2.57718300000000     -0.62814100000000      3.91746800000000
Ni  0.02052800000000     -0.01214800000000      8.35110000000000
O   0.13190100000000      2.07209900000000      8.37771100000000
O  -0.09084400000000     -2.09639500000000      8.32448800000000
O  -2.06388400000000      0.09926800000000      8.34841300000000
O   2.10494100000000     -0.12356300000000      8.35378700000000
O   0.01642500000000     -0.03857800000000     10.43831800000000
H  -0.54404800000000      2.59824300000000      8.83883900000000
H  -0.02409600000000      2.16166300000000      7.40756400000000
H   0.79793500000000     -2.38740200000000      8.60424700000000
H  -0.02755200000000     -1.96685100000000      7.34068400000000
H  -2.41721400000000      0.95350100000000      8.04262800000000
H  -1.97217200000000      0.16346900000000      9.33395400000000
H   2.38466700000000      0.71248000000000      8.77157900000000
H   2.04281500000000      0.06940700000000      7.38311000000000
Ni  0.01232200000000     -0.06500800000000     12.52553700000000
O   0.12369500000000      2.01923900000000     12.55214800000000
O  -0.09905000000000     -2.14925500000000     12.49892600000000
O  -2.07209100000000      0.04640700000000     12.52285000000000
O   2.09673500000000     -0.17642400000000     12.52822400000000
O   0.00821900000000     -0.09143800000000     14.61275600000000
H   0.18414300000000      0.74582900000000     15.07853100000000
H   0.63714700000000     -0.73845000000000     14.98280400000000
H   0.12170700000000      1.87545100000000     11.55963200000000
H  -0.74610800000000      2.41427500000000     12.75483800000000
H  -0.89278600000000     -2.64417300000000     12.77005100000000
H  -0.11720000000000     -2.10178600000000     11.50772600000000
H  -2.63365300000000     -0.60027200000000     12.98762300000000
H  -2.05402400000000     -0.18737500000000     11.56160400000000
H   2.51568100000000      0.68694800000000     12.70603100000000
H   2.06785300000000     -0.25691100000000     11.54147900000000
```

v. 5 Ni:

```
Ni  0.12893400000000      0.03779500000000      0.00112800000000
O   2.21517600000000     -0.02364300000000      0.03304300000000
O  -1.95730700000000      0.09923200000000     -0.03078800000000
O   0.19021900000000      2.12425900000000      0.01153000000000
O   0.06764900000000     -2.04867000000000     -0.00927500000000
O   0.16114200000000      0.04725500000000     -2.08599300000000
O   0.09672700000000      0.02833500000000      2.08824800000000
H   2.08131100000000     -0.01020000000000      1.02236000000000
H   2.52119700000000     -0.93036300000000     -0.16287700000000
H  -0.68844700000000      2.51529500000000     -0.15503000000000
```

```
H   0.24299300000000      2.02334900000000      1.00223900000000
H  -1.82524200000000      0.08916400000000      0.96422000000000
H  -2.48394900000000     -0.69128300000000     -0.24736600000000
H  -0.09252700000000     -2.02488800000000      0.96929100000000
H  -0.61807700000000     -2.59883300000000     -0.42703400000000
H  -0.56607700000000      0.51881600000000     -2.53059200000000
H   0.97973400000000      0.46068400000000     -2.41845300000000
Ni  0.06451900000000      0.01887600000000      4.17536800000000
O   0.12580400000000      2.10534000000000      4.18577000000000
O   0.00323400000000     -2.06758800000000      4.16496600000000
O   0.03231100000000      0.00941700000000      6.26248800000000
O   2.15076000000000     -0.04256100000000      4.20728300000000
O  -2.02172300000000      0.08031300000000      4.14345200000000
H  -0.70220500000000      2.33566200000000      3.72381100000000
H  -0.10615000000000      2.03716200000000      5.14677800000000
H   0.19136400000000     -2.05736600000000      5.13593900000000
H   0.84852100000000     -2.28546400000000      3.72904300000000
H   2.08295600000000      0.18037800000000      5.16956300000000
H   2.39784300000000      0.78585400000000      3.75432800000000
H  -2.00806700000000     -0.10485800000000      5.11589800000000
H  -2.24128500000000     -0.76728300000000      3.71423600000000
Ni  0.00010400000000     -0.00004300000000      8.34960800000000
O   0.06138900000000      2.08642100000000      8.36001000000000
O  -0.06118100000000     -2.08650700000000      8.33920600000000
O  -2.08613800000000      0.06139500000000      8.31769200000000
O   2.08634500000000     -0.06148000000000      8.38152300000000
O  -0.03210400000000     -0.00950200000000     10.43672800000000
H   0.87059100000000      2.26962600000000      8.87358200000000
H   0.34380700000000      2.03106200000000      7.41367500000000
H  -0.34335900000000     -2.02929800000000      9.28560600000000
H  -0.87078900000000     -2.26910000000000      7.82604600000000
H  -2.31156900000000      0.88347100000000      7.84423000000000
H  -2.01500500000000      0.31207900000000      9.27358900000000
H   2.31002400000000     -0.88498100000000      8.85333600000000
H   2.01318500000000     -0.31083500000000      7.42530300000000
Ni -0.06431100000000     -0.01896200000000     12.52384800000000
O  -0.00302700000000      2.06750300000000     12.53425000000000
O  -0.12559600000000     -2.10542600000000     12.51344600000000
O  -2.15055300000000      0.04247600000000     12.49193200000000
O   2.02193000000000     -0.08039900000000     12.55576400000000
O  -0.09651900000000     -0.02842100000000     14.61096800000000
H  -0.18926400000000      2.05556500000000     11.56284400000000
H  -0.84912300000000      2.28637800000000     12.96806500000000
H   0.70138800000000     -2.34020800000000     12.97489600000000
H   0.10583300000000     -2.03894500000000     11.55227100000000
H  -2.39571000000000     -0.78663200000000     12.94469800000000
H  -2.08109600000000     -0.18030100000000     11.52962500000000
H   2.24348600000000      0.76660900000000     12.98504200000000
H   2.00978800000000      0.10449100000000     11.58334900000000
Ni -0.12872600000000     -0.03788000000000     16.69808800000000
O  -0.06744200000000      2.04858400000000     16.70849000000000
O  -0.19001100000000     -2.12434400000000     16.68768600000000
O  -2.21496800000000      0.02355700000000     16.66617300000000
O   1.95751500000000     -0.09931700000000     16.73000400000000
O  -0.16093400000000     -0.04733900000000     18.78520800000000
H  -0.98005400000000     -0.45975500000000     19.11763600000000
H   0.56598000000000     -0.51904400000000     19.23016800000000
H   0.61526100000000      2.60188500000000     17.12707200000000
H   0.09279200000000      2.02666700000000     15.72999600000000
H   0.68864000000000     -2.51535500000000     16.85440400000000
H  -0.24186700000000     -2.02249800000000     15.69692900000000
H  -2.52146100000000      0.93005900000000     16.86239400000000
H  -2.08197600000000      0.01010500000000     15.67678400000000
H   1.82176200000000     -0.08850900000000     15.73511600000000
H   2.48162500000000      0.69296400000000     16.94614599999999
```

vi. 6 Ni:

```
Ni -0.08614600000000      0.07092100000000      0.00057800000000
O   2.00118500000000      0.07094800000000     -0.01512100000000
O  -2.17347700000000      0.07089500000000      0.01627700000000
O  -0.08605400000000      2.15825200000000      0.01628700000000
O  -0.08623700000000     -2.01641000000000     -0.01513000000000
O  -0.10184500000000      0.08663000000000     -2.08669400000000
O  -0.07044700000000      0.05521200000000      2.08785000000000
H   1.84846900000000      0.06862600000000      0.97873400000000
H   2.50589500000000     -0.73767500000000     -0.21614800000000
H   0.80877900000000      2.51907600000000     -0.13096800000000
H  -0.15376100000000      2.04347800000000      1.00626800000000
H  -2.03871200000000      0.08143100000000      1.00674400000000
H  -2.50052700000000     -0.82883200000000     -0.17745600000000
H   0.04963000000000     -1.97439900000000      0.96892500000000
H   0.60813800000000     -2.57890400000000     -0.40080600000000
H  -0.90675200000000      0.52878500000000     -2.41543000000000
H   0.64053000000000      0.55086500000000     -2.51348000000000
Ni -0.05474700000000      0.03950400000000      4.17512200000000
O  -0.05465600000000      2.12683500000000      4.19083000000000
O  -0.05483900000000     -2.04782700000000      4.15941300000000
O  -0.03904800000000      0.02379500000000      6.26239400000000
O   2.03258400000000      0.03953000000000      4.15942200000000
O  -2.14207800000000      0.03947700000000      4.19082100000000
H   0.11106700000000      2.04166000000000      5.16730700000000
H   0.80846600000000      2.33903500000000      3.78970700000000
H  -0.92479600000000     -2.24413100000000      3.76418100000000
H  -0.21059900000000     -1.97384500000000      5.13901200000000
H   1.98068800000000     -0.12466100000000      5.13667400000000
H   2.22492500000000     -0.82697100000000      3.75580600000000
H  -2.03965200000000      0.22586200000000      5.16124200000000
H  -2.35358500000000      0.89507800000000      3.77190100000000
Ni -0.02334900000000      0.00808700000000      8.34966500000000
O  -0.02325700000000      2.09541800000000      8.36537400000000
O  -0.02344100000000     -2.07924400000000      8.33395700000000
O  -2.11068000000000      0.00806000000000      8.36536500000000
O   2.06398200000000      0.00811300000000      8.33396600000000
O  -0.00765000000000     -0.00762200000000     10.43693700000000
H  -0.28693500000000      1.99832300000000      9.31637900000000
H  -0.83453000000000      2.34108600000000      7.88342400000000
H   0.23682800000000     -2.07214100000000      9.28713300000000
H   0.78963300000000     -2.26415600000000      7.82779500000000
H  -2.00560800000000     -0.23180400000000      7.40688600000000
H  -2.35734000000000     -0.81860300000000      8.81924300000000
H   1.99438200000000      0.23268700000000      9.29713300000000
H   2.24727200000000      0.84880000000000      7.87370500000000
Ni  0.00804900000000     -0.02333100000000     12.52420900000000
O   0.00814100000000      2.06400000000000     12.53991700000000
O   0.00795800000000     -2.11066100000000     12.50850100000000
O  -2.07928200000000     -0.02335700000000     12.53990900000000
O   2.09538000000000     -0.02330400000000     12.50851000000000
O   0.02374800000000     -0.03903900000000     14.61148100000000
H   0.84847800000000      2.24771800000000     13.00063600000000
H   0.23304800000000      1.99529100000000     11.57682200000000
H  -0.81850500000000     -2.35788200000000     12.05457100000000
H  -0.23210600000000     -2.00571400000000     13.46694100000000
H  -2.26364100000000      0.78972300000000     13.04626900000000
H  -2.07188800000000      0.23722100000000     11.58681400000000
H   2.34071200000000     -0.83568000000000     12.98877800000000
H   1.99772600000000     -0.28533100000000     11.55704900000000
```

```
Ni  0.03944800000000    -0.05474800000000    16.69875300000000
O   0.03953900000000     2.03258300000000    16.71446100000000
O   0.03935600000000    -2.14207900000000    16.68304500000000
O  -2.04788300000000    -0.05477400000000    16.71445200000000
O   2.12677900000000    -0.05472100000000    16.68305300000000
O   0.05514700000000    -0.07045600000000    18.78602500000000
H  -0.82722500000000     2.22389500000000    17.11805099999999
H  -0.12446700000000     1.98069100000000    15.73717000000000
H   0.89472400000000    -2.35342600000000    17.10254600000000
H   0.22640600000000    -2.04031200000000    15.71272600000000
H  -1.97338700000000    -0.21040900000000    15.73482600000000
H  -2.24227200000000    -0.92520300000000    17.10965000000000
H   2.33823800000000     0.80781100000000    17.08588700000000
H   2.04354800000000     0.11217700000000    15.70675600000000
Ni  0.07084600000000    -0.08616500000000    20.87329700000000
O   0.07093800000000     2.00116600000000    20.88900500000000
O   0.07075400000000    -2.17349600000000    20.85758800000000
O  -2.01648500000000    -0.08619200000000    20.88899599999999
O   2.15817700000000    -0.08613800000000    20.85759699999999
O   0.08654600000000    -0.10187400000000    22.96056800000000
H   0.55080900000000     0.64038200000000    23.38751300000000
H   0.52851100000000    -0.90686300000000    23.28933400000000
H   0.06861200000000     1.84824100000000    19.89516800000000
H  -0.73747900000000     2.50620600000000    21.08997000000000
H  -0.82895500000000    -2.50064000000000    21.05119600000000
H   0.08123600000000    -2.03792300000000    19.86717800000000
H  -2.57827200000000     0.60866900000000    21.27480300000000
H  -1.97441300000000     0.04982900000000    19.90498100000000
H   2.51878400000000     0.80880500000000    21.00467300000000
H   2.04291700000000    -0.15393100000000    19.86765300000000
```

vii. 7 Ni:

```
Ni -0.06894700000000     0.12001500000000    -0.00106500000000
O   2.01841500000000     0.12609000000000    -0.00995100000000
O  -2.15630900000000     0.11394100000000     0.00782100000000
O  -0.07493500000000     2.20730000000000     0.01908600000000
O  -0.06295800000000    -1.96726900000000    -0.02121700000000
O  -0.07789100000000     0.14014100000000    -2.08833900000000
O  -0.06000200000000     0.09989000000000     2.08620900000000
H   1.83730500000000     0.13348500000000     0.97972900000000
H   2.35189400000000    -0.77186700000000    -0.19784300000000
H   0.81662900000000     2.58000800000000    -0.11821900000000
H  -0.14220400000000     2.06002800000000     1.00352400000000
H  -2.07704600000000     0.16478300000000     0.99924300000000
H  -2.67460500000000    -0.68782500000000    -0.18246300000000
H  -0.21827600000000    -1.91269100000000     0.95798300000000
H  -0.78082300000000    -2.49621700000000    -0.41176300000000
H  -0.71197500000000     0.80867200000000    -2.40812500000000
H   0.77415200000000     0.36650900000000    -2.50257800000000
Ni -0.05105800000000     0.07976400000000     4.17348200000000
O  -0.05704600000000     2.16704800000000     4.19363400000000
O  -0.04507000000000    -2.00752000000000     4.15333100000000
O  -0.04211400000000     0.05963900000000     6.26075600000000
O   2.03630400000000     0.08583900000000     4.16459600000000
O  -2.13842000000000     0.07369000000000     4.18236900000000
H   0.85098700000000     2.42599700000000     4.43619200000000
H  -0.04339200000000     2.06623600000000     3.20661000000000
H   0.01165600000000    -1.90743600000000     5.14713200000000
H   0.76777100000000    -2.45710900000000     3.86448900000000
H   1.98182800000000    -0.06420500000000     5.14626200000000
H   2.49226100000000    -0.67948300000000     3.77466500000000
H  -2.00995400000000     0.17072600000000     5.16537600000000
H  -2.37392400000000    -0.86355400000000     4.05081400000000
```

```
Ni -0.03316900000000      0.03951300000000      8.34803000000000
O  -0.03915800000000      2.12679800000000      8.36818100000000
O  -0.02718100000000     -2.04777100000000      8.32787900000000
O  -2.12053100000000      0.03343900000000      8.35691600000000
O   2.05419300000000      0.04558800000000      8.33914400000000
O  -0.02422500000000      0.01938800000000     10.43530400000000
H  -0.85108500000000      2.29706000000000      8.88118700000000
H  -0.30260300000000      2.13059900000000      7.41536100000000
H   0.14541600000000     -1.97237000000000      9.30501800000000
H   0.83997500000000     -2.22099500000000      7.91701500000000
H  -2.04273900000000     -0.25386000000000      7.41249200000000
H  -2.34236500000000     -0.76416700000000      8.87148700000000
H   1.93174600000000      0.17873100000000      9.32128000000000
H   2.27269100000000      0.92825300000000      7.98808600000000
Ni -0.01528000000000     -0.00073700000000     12.52257800000000
O  -0.02126900000000      2.08654700000000     12.54272900000000
O  -0.00929200000000     -2.08802200000000     12.50242700000000
O  -2.10264300000000     -0.00681200000000     12.53146400000000
O   2.07208200000000      0.00533700000000     12.51369100000000
O  -0.00633600000000     -0.02086300000000     14.60985200000000
H   0.82502700000000      2.31468400000000     12.96863800000000
H   0.17786000000000      2.00826500000000     11.57177400000000
H  -0.86152400000000     -2.31757500000000     12.08933200000000
H  -0.19461800000000     -2.01402000000000     13.47660800000000
H  -2.27654200000000      0.81754000000000     13.02312300000000
H  -2.04245200000000      0.24242300000000     11.57307200000000
H   2.00441200000000     -0.24187400000000     13.47270100000000
H   2.24330600000000     -0.82140400000000     12.02505700000000
Ni  0.00260900000000     -0.04098800000000     16.69712500000000
O  -0.00338000000000      2.04629600000000     16.71727700000000
O   0.00859700000000     -2.12827200000000     16.67697400000000
O  -2.08475400000000     -0.04706300000000     16.70601200000000
O   2.08997100000000     -0.03491400000000     16.68823900000000
O   0.01155300000000     -0.06111400000000     18.78439900000000
H  -0.87245100000000      2.21783000000000     17.12514700000000
H  -0.17490000000000      1.96565500000000     15.73985000000000
H   0.17022600000000     -2.10247800000000     17.65397500000000
H   0.87091900000000     -2.30839900000000     16.25908400000000
H  -1.96799300000000     -0.19081200000000     15.72542800000000
H  -2.32401300000000     -0.92030000000000     17.06759700000000
H   2.01291200000000      0.27659100000000     17.62465400000000
H   2.30266100000000      0.74881600000000     16.14869800000000
Ni  0.02049800000000     -0.08123900000000     20.87167300000000
O   0.01450900000000      2.00604500000000     20.89182400000000
O   0.02648600000000     -2.16852300000000     20.85152200000000
O  -2.06686500000000     -0.08731300000000     20.88055900000000
O   2.10786000000000     -0.07516500000000     20.86278699999999
O   0.02944200000000     -0.10136400000000     22.95894700000000
H  -0.05358700000000      1.91363100000000     19.89895599999999
H  -0.80321900000000      2.43511100000000     21.19745099999999
H  -0.00080600000000     -2.06422100000000     21.83833300000000
H   0.94627900000000     -2.43027200000000     20.65925199999999
H  -2.52866700000000      0.74238300000000     21.09160600000000
H  -1.98193500000000     -0.11028100000000     19.88924900000000
H   2.32927900000000      0.81778600000000     21.18572400000000
H   1.95950800000000      0.02207500000000     19.87908400000000
```

```
Ni  0.03838600000000     -0.12149000000000     25.04622099999999
O   0.03239800000000      1.96579400000000     25.06637200000000
O   0.04437500000000     -2.20877400000000     25.02607000000000
O  -2.04897600000000     -0.12756400000000     25.05510700000000
O   2.12574900000000     -0.11541500000000     25.03733499999999
O   0.04733100000000     -0.14161500000000     27.13349400000000
H   0.74548700000000      2.50519000000000     25.45136800000000
H   0.17856100000000      1.91361300000000     24.08582199999999
H  -0.84524500000000     -2.58317600000000     25.17301300000000
H   0.09409800000000     -2.05420700000000     24.04120000000000
H  -2.39175800000000      0.76760500000000     25.23879799999999
H  -1.89889800000000     -0.14792800000000     24.06347400000000
H   2.01539100000000     -0.15083200000000     24.04612500000000
H   2.63001100000000      0.69552700000000     25.22712200000000
H  -0.80752800000000     -0.35811000000000     27.54730600000000
H   0.67392800000000     -0.81643400000000     27.45460100000000
```

viii. 8 Ni:

```
Ni -0.08238700000000      0.06788100000000      0.00048100000000
O   2.00497000000000      0.06308200000000     -0.01028300000000
O  -2.16974400000000      0.07267900000000      0.01124400000000
O  -0.07753400000000      2.15523800000000      0.01118100000000
O  -0.08724000000000     -2.01947600000000     -0.01021900000000
O  -0.09312600000000      0.07860600000000     -2.08685400000000
O  -0.07164800000000      0.05715600000000      2.08781500000000
H   1.83476700000000      0.06593600000000      0.98239100000000
H   2.51256700000000     -0.74748100000000     -0.19538600000000
H   0.82240200000000      2.50964100000000     -0.11886100000000
H  -0.15367600000000      2.02437800000000      1.00013600000000
H  -2.02224400000000      0.09177900000000      1.00125800000000
H  -2.49304600000000     -0.83107000000000     -0.16855600000000
H   0.02927400000000     -1.94241200000000      0.97698200000000
H   0.63170200000000     -2.57510800000000     -0.35889900000000
H  -0.90018000000000      0.52773800000000     -2.40046400000000
H   0.64624600000000      0.56358400000000     -2.49527200000000
Ni -0.06090900000000      0.04643100000000      4.17515000000000
O  -0.05605600000000      2.13378800000000      4.18585000000000
O  -0.06576300000000     -2.04092600000000      4.16445000000000
O  -0.05017100000000      0.03570600000000      6.26248500000000
O   2.02644700000000      0.04163300000000      4.16438700000000
O  -2.14826600000000      0.05122900000000      4.18591400000000
H   0.09311800000000      2.02597500000000      5.16538300000000
H   0.81859600000000      2.33093300000000      3.80243000000000
H  -0.94551500000000     -2.22270800000000      3.78437000000000
H  -0.20807600000000     -1.93539000000000      5.14610200000000
H   1.91552600000000     -0.09374500000000      5.14734000000000
H   2.21107600000000     -0.84236500000000      3.79684200000000
H  -2.04233000000000      0.20542700000000      5.16441900000000
H  -2.35134200000000      0.92202200000000      3.79604400000000
Ni -0.03943200000000      0.02498200000000      8.34982000000000
O  -0.03457800000000      2.11233900000000      8.36052000000000
O  -0.04428500000000     -2.06237500000000      8.33912000000000
O  -2.12678800000000      0.02978000000000      8.36058400000000
O   2.04792500000000      0.02018300000000      8.33905600000000
O  -0.02869300000000      0.01425700000000     10.43715500000000
H  -0.29947800000000      2.02660600000000      9.31205100000000
H  -0.85574500000000      2.27259700000000      7.85786300000000
H   0.20881500000000     -2.04243200000000      9.29478600000000
H   0.77773300000000     -2.21426500000000      7.83585700000000
H  -2.28277600000000     -0.77326600000000      7.82789900000000
H  -2.05619300000000     -0.26435900000000      9.30328000000000
H   2.01335800000000      0.24441800000000      9.30277900000000
H   2.20627400000000      0.85869000000000      7.86619800000000
```

```
Ni -0.01795400000000      0.00353200000000     12.52449000000000
O  -0.01310100000000      2.09088900000000     12.53519000000000
O  -0.02280800000000     -2.08382500000000     12.51379000000000
O  -2.10531100000000      0.00833000000000     12.53525300000000
O   2.06940300000000     -0.00126600000000     12.51372600000000
O  -0.00721500000000     -0.00719300000000     14.61182400000000
H   0.75643500000000      2.29613700000000     13.09794100000000
H   0.32458400000000      2.02875300000000     11.60721500000000
H  -0.87828800000000     -2.29455800000000     12.09658700000000
H  -0.21624500000000     -1.97809500000000     13.48459200000000
H  -2.23075900000000      0.74245200000000     13.16737800000000
H  -2.08489900000000      0.40671100000000     11.63336000000000
H   1.98322900000000     -0.21241200000000     13.48145600000000
H   2.24910900000000     -0.84855900000000     12.06506700000000
Ni  0.00352400000000     -0.01791800000000     16.69915900000000
O   0.00837700000000      2.06943900000000     16.70985900000000
O  -0.00133000000000     -2.10527400000000     16.68845900000000
O  -2.08383300000000     -0.01311900000000     16.70992300000000
O   2.09088000000000     -0.02271600000000     16.68839600000000
O   0.01426200000000     -0.02864200000000     18.78649400000000
H  -0.83807400000000      2.25355500000000     17.15831700000000
H  -0.20282500000000      1.98489700000000     15.74202800000000
H   0.40015000000000     -2.08796000000000     17.58895700000000
H   0.73005800000000     -2.23228800000000     16.05341500000000
H  -1.97921000000000     -0.20641400000000     15.73899100000000
H  -2.29609600000000     -0.86835100000000     17.12689800000000
H   2.02996300000000      0.31603400000000     17.61603599999999
H   2.29914500000000      0.74546100000000     16.12491700000000
Ni  0.02500100000000     -0.03936700000000     20.87382900000000
O   0.02985500000000      2.04799000000000     20.88452899999999
O   0.02014800000000     -2.12672400000000     20.86312900000000
O  -2.06235500000000     -0.03456900000000     20.88459200000000
O   2.11235800000000     -0.04416500000000     20.86306499999999
O   0.03574000000000     -0.05009200000000     22.96116400000000
H   0.86945700000000      2.20340900000000     21.35639099999999
H   0.25325300000000      2.01032700000000     19.92058900000000
H  -0.27391700000000     -2.05325400000000     19.92054600000000
H  -0.78383200000000     -2.27835200000000     21.39567900000000
H  -2.21217100000000      0.78763300000000     21.38815000000000
H  -2.04146100000000      0.21880500000000     19.92903200000000
H   2.26985500000000     -0.86581500000000     21.36580500000000
H   2.02644800000000     -0.30911000000000     19.91160200000000
Ni  0.04647900000000     -0.06081700000000     25.04849900000000
O   0.05133200000000      2.02654000000000     25.05919800000000
O   0.04162500000000     -2.14817400000000     25.03779900000000
O  -2.04087800000000     -0.05601800000000     25.05926200000000
O   2.13383600000000     -0.06561500000000     25.03773500000000
O   0.05721700000000     -0.07154100000000     27.13583300000000
H  -0.83170700000000      2.21572000000000     25.42672300000000
H  -0.08452900000000      1.91677800000000     24.07623399999999
H   0.91166900000000     -2.35490400000000     25.42744899999999
H   0.19589300000000     -2.04328500000000     24.05918899999999
H  -1.93478200000000     -0.19860100000000     24.07762599999999
H  -2.22657500000000     -0.93517900000000     25.43885600000000
H   2.33643600000000      0.80886200000000     25.41863299999999
H   2.02506200000000      0.08189000000000     24.05787300000000
```

```
Ni  0.06795600000000     -0.08226600000000     29.22316800000000
O   0.07280900000000      2.00509100000000     29.23386800000000
O   0.06310200000000     -2.16962300000000     29.21246799999999
O  -2.01940100000000     -0.07746800000000     29.23393099999999
O   2.15531300000000     -0.08706400000000     29.21240500000000
O   0.07869400000000     -0.09299100000000     31.31050299999999
H   0.07467600000000      1.83484600000000     28.24122400000000
H  -0.73539200000000      2.51627300000000     29.41946000000000
H  -0.84260900000000     -2.48784200000000     29.39153499999999
H   0.08404700000000     -2.02266900000000     28.22244900000000
H  -2.57220900000000      0.64463300000000     29.58055200000000
H  -1.94004500000000      0.03701100000000     28.24648000000000
H   2.51430500000000      0.81082300000000     29.34400800000000
H   2.02376900000000     -0.16092000000000     28.22328699999999
H   0.56416600000000      0.64568600000000     31.71961700000000
H   0.52661800000000     -0.90081600000000     31.62390600000000
```

ix. 9 Ni:

```
Ni -0.08688900000000      0.10264100000000     -0.00183700000000
O   2.00046900000000      0.10729600000000     -0.01237200000000
O  -2.17424700000000      0.09798500000000      0.00869800000000
O  -0.09148900000000      2.18999700000000      0.00922000000000
O  -0.08228900000000     -1.98471500000000     -0.01289400000000
O  -0.09744900000000      0.11367400000000     -2.08917100000000
O  -0.07633000000000      0.09160700000000      2.08549700000000
H   1.80653100000000      0.11810800000000      0.97568700000000
H   2.33717200000000     -0.79097600000000     -0.19130600000000
H   0.80765800000000      2.54943500000000     -0.11232800000000
H  -0.16984900000000      2.03226100000000      0.99231900000000
H  -2.08002400000000      0.15263000000000      0.99924200000000
H  -2.70940500000000     -0.69571900000000     -0.16843000000000
H  -0.22277600000000     -1.89975100000000      0.96819800000000
H  -0.82501500000000     -2.49868100000000     -0.37572100000000
H  -0.72512400000000      0.79241700000000     -2.39961600000000
H   0.75895400000000      0.34618300000000     -2.49061700000000
Ni -0.06577000000000      0.08057400000000      4.17283200000000
O  -0.07037000000000      2.16793000000000      4.18388800000000
O  -0.06117000000000     -2.00678200000000      4.16177500000000
O  -0.05521100000000      0.06954000000000      6.26016600000000
O   2.02158800000000      0.08522900000000      4.16229700000000
O  -2.15312800000000      0.07591800000000      4.18336700000000
H   0.82893700000000      2.41941400000000      4.46370200000000
H  -0.01906200000000      2.06167300000000      3.19868200000000
H  -0.00440800000000     -1.88800700000000      5.15553100000000
H   0.75366100000000     -2.45782200000000      3.88092000000000
H   1.95208800000000     -0.05993800000000      5.14530900000000
H   2.46433000000000     -0.69200100000000      3.78086600000000
H  -2.01497600000000      0.17577800000000      5.16592100000000
H  -2.38384300000000     -0.86316100000000      4.05677400000000
Ni -0.04465100000000      0.05850700000000      8.34750000000000
O  -0.04925100000000      2.14586300000000      8.35855700000000
O  -0.04005100000000     -2.02884900000000      8.33644300000000
O  -2.13200900000000      0.05385100000000      8.35803500000000
O   2.04270700000000      0.06316200000000      8.33696500000000
O  -0.03409200000000      0.04747300000000     10.43483400000000
H  -0.84297800000000      2.29546000000000      8.90585900000000
H  -0.34876300000000      2.14156800000000      7.41688200000000
H   0.12670800000000     -1.92758700000000      9.31457300000000
H   0.83289800000000     -2.19175000000000      7.93361800000000
H  -2.06152700000000     -0.28759900000000      7.43237000000000
H  -2.32693600000000     -0.71296300000000      8.92806600000000
H   1.91101400000000      0.19947400000000      9.31854800000000
H   2.24595000000000      0.94818600000000      7.98253500000000
```

```
Ni -0.02353200000000     0.03644000000000    12.52216800000000
O  -0.02813200000000     2.12379600000000    12.53322500000000
O  -0.01893200000000    -2.05091600000000    12.51111100000000
O  -2.11089000000000     0.03178400000000    12.53270300000000
O   2.06382600000000     0.04109600000000    12.51163300000000
O  -0.01297300000000     0.02540600000000    14.60950200000000
H   0.81549900000000     2.34361000000000    12.96874300000000
H   0.18095200000000     2.02866600000000    11.56484000000000
H  -0.87483800000000    -2.26424200000000    12.09681600000000
H  -0.20656400000000    -1.95305000000000    13.48411600000000
H  -2.26223300000000     0.84896500000000    13.04414700000000
H  -2.04240600000000     0.29986500000000    11.57971500000000
H   1.99573900000000    -0.19441400000000    13.47411400000000
H   2.22181700000000    -0.79335800000000    12.03168200000000
Ni -0.00241300000000     0.01437300000000    16.69683600000000
O  -0.00701300000000     2.10172900000000    16.70789300000000
O   0.00218700000000    -2.07298300000000    16.68577900000000
O  -2.08977100000000     0.00971700000000    16.70737100000000
O   2.08494500000000     0.01902900000000    16.68630100000000
O   0.00814600000000     0.00334000000000    18.78417000000000
H  -0.83193900000000     2.25468600000000    17.20604300000000
H  -0.25901700000000     2.04548500000000    15.75005300000000
H   0.25369500000000    -2.01659000000000    17.64378400000000
H   0.82763300000000    -2.22429100000000    16.18796600000000
H  -1.99167500000000    -0.18446100000000    15.73570400000000
H  -2.30628500000000    -0.84161500000000    17.12929199999999
H   1.98686400000000     0.21085800000000    17.65850900000000
H   2.30095200000000     0.87164600000000    16.26675100000000
Ni  0.01870600000000    -0.00769400000000    20.87150500000000
O   0.01410600000000     2.07966200000000    20.88256100000000
O   0.02330600000000    -2.09505000000000    20.86044799999999
O  -2.06865200000000    -0.01235000000000    20.88204000000000
O   2.10606400000000    -0.00303800000000    20.86097000000000
O   0.02926500000000    -0.01872700000000    22.95883900000000
H   0.86802300000000     2.29375200000000    21.30050600000000
H   0.20467900000000     1.98408200000000    19.91013300000000
H  -0.82312800000000    -2.31291200000000    20.42939600000000
H  -0.18170600000000    -1.99697600000000    21.82977300000000
H  -2.22758600000000     0.81877200000000    21.36751200000000
H  -2.00342800000000     0.22845400000000    19.92098099999999
H   2.03584800000000    -0.26201700000000    21.81673700000000
H   2.25890800000000    -0.82520800000000    20.35816000000000
Ni  0.03982500000000    -0.02976100000000    25.04617300000000
O   0.03522500000000     2.05759500000000    25.05723000000000
O   0.04442500000000    -2.11711700000000    25.03511600000000
O  -2.04753300000000    -0.03441600000000    25.05670800000000
O   2.12718300000000    -0.02510500000000    25.03563800000000
O   0.05038400000000    -0.04079400000000    27.13350699999999
H  -0.82387500000000     2.22275400000000    25.48872500000000
H  -0.15552300000000     1.96755900000000    24.08379699999999
H   0.33501600000000    -2.11044700000000    25.97994600000000
H   0.84328100000000    -2.26628700000000    24.49523900000000
H  -1.91914200000000    -0.17408900000000    24.07571000000000
H  -2.26125300000000    -0.91570000000000    25.41389100000000
H   2.02449400000000     0.27598900000000    25.97501900000000
H   2.31890700000000     0.77014900000000    24.50506300000000
Ni  0.06094400000000    -0.05182800000000    29.22084099999999
O   0.05634400000000     2.03552800000000    29.23189800000000
O   0.06554400000000    -2.13918300000000    29.20978400000000
O  -2.02641400000000    -0.05648300000000    29.23137599999999
O   2.14830200000000    -0.04717200000000    29.21030599999999
O   0.07150300000000    -0.06286100000000    31.30817499999999
```

```
H   0.85323900000000     2.46125100000000     29.59253799999999
H   0.18257500000000     1.94584100000000     28.24505900000000
H  -0.84984500000000    -2.37507700000000     28.97133699999999
H   0.05704400000000    -2.01659600000000     30.19569600000000
H  -2.22744100000000     0.81012500000000     29.63071800000000
H  -1.94614800000000     0.10580100000000     28.25277000000000
H   2.60191300000000     0.80682600000000     29.32087900000000
H   1.99808700000000    -0.15107400000000     28.22734600000000
Ni  0.08206300000000    -0.07389400000000     33.39550900000000
O   0.07746300000000     2.01346200000000     33.40656600000000
O   0.08666300000000    -2.16124900000000     33.38445300000000
O  -2.00529500000000    -0.07854900000000     33.40604500000000
O   2.16942100000000    -0.06923800000000     33.38497400000000
O   0.09262300000000    -0.08492600000000     35.48284400000000
H  -0.81139500000000     2.37779000000000     33.57780700000000
H   0.10149100000000     1.87842800000000     32.41814900000000
H  -0.63007800000000    -2.73779000000000     33.70323600000000
H  -0.01919700000000    -2.05188700000000     32.40151000000000
H  -2.52148300000000    -0.88084100000000     33.60362300000000
H  -1.82964700000000    -0.09965600000000     32.41612600000000
H   2.49755100000000    -0.97120300000000     33.56434700000000
H   2.03618600000000    -0.04975300000000     32.39548200000000
H   0.89676500000000     0.36773600000000     35.79837000000000
H  -0.64981800000000     0.39328800000000     35.89328200000000
```

x. 10 Ni:

```
Ni -0.08067600000000    -0.03164800000000    -0.05728200000000
O   2.00656300000000    -0.05422400000000    -0.06839200000000
O  -2.16791400000000    -0.00907300000000    -0.04617200000000
O  -0.05811700000000     2.05561700000000    -0.06041500000000
O  -0.10323400000000    -2.11891400000000    -0.05415100000000
O  -0.09181900000000    -0.03466000000000    -2.14464000000000
O  -0.06953300000000    -0.02863700000000     2.03007600000000
H   2.52074600000000     0.75502800000000    -0.24047700000000
H   1.82096900000000    -0.05898700000000     0.92310400000000
H   0.67937700000000     2.60479800000000    -0.37936900000000
H   0.04218400000000     1.94960000000000     0.92777800000000
H  -2.47517300000000     0.90239200000000    -0.21395900000000
H  -2.00821200000000    -0.03791700000000     0.94253700000000
H  -0.17808000000000    -1.97441000000000     0.93414400000000
H   0.79437600000000    -2.48172500000000    -0.17609200000000
H  -0.90437900000000    -0.48294100000000    -2.44500400000000
H   0.64091600000000    -0.54056900000000    -2.53935600000000
Ni -0.05839000000000    -0.02562500000000     4.11743400000000
O  -0.03583100000000     2.06164100000000     4.11430200000000
O  -0.08094800000000    -2.11289100000000     4.12056600000000
O  -0.04724600000000    -0.02261300000000     6.20479200000000
O   2.02884900000000    -0.04820000000000     4.10632400000000
O  -2.14562800000000    -0.00305000000000     4.12854400000000
H  -0.17506600000000     1.94872900000000     5.09648800000000
H  -0.91672700000000     2.24695900000000     3.73868900000000
H   0.79552900000000    -2.31282700000000     3.74305800000000
H   0.06290100000000    -2.00303400000000     5.10191900000000
H   1.90448500000000     0.07742000000000     5.09099100000000
H   2.21724900000000     0.83969500000000     3.75053700000000
H  -2.03251300000000    -0.14997100000000     5.10884200000000
H  -2.35274000000000    -0.87648000000000     3.74712400000000
Ni -0.03610300000000    -0.01960200000000     8.29215000000000
O  -0.01354500000000     2.06766400000000     8.28901800000000
O  -0.05866200000000    -2.10686800000000     8.29528200000000
O  -2.12334200000000     0.00297300000000     8.30326000000000
O   2.05113500000000    -0.04217700000000     8.28104000000000
O  -0.02496000000000    -0.01659000000000    10.37950800000000
```

```
H   0.19221300000000      1.98019900000000      9.25792300000000
H   0.83939700000000      2.20950900000000      7.83731000000000
H  -0.25367400000000     -2.01745700000000      9.26611900000000
H  -0.91576800000000     -2.25697600000000      7.85427200000000
H  -2.02057300000000      0.20285700000000      9.27256800000000
H  -2.27322700000000      0.85835100000000      7.85885200000000
H   2.19292900000000     -0.90084700000000      7.84034100000000
H   1.95583400000000     -0.23648100000000      9.25265100000000
Ni -0.01381700000000     -0.01357900000000     12.46686600000000
O   0.00874100000000      2.07368700000000     12.46373400000000
O  -0.03637600000000     -2.10084400000000     12.46999800000000
O  -2.10105600000000      0.00899600000000     12.47797600000000
O   2.07342100000000     -0.03615400000000     12.45575600000000
O  -0.00267400000000     -0.01056700000000     14.55422400000000
H  -0.20446700000000      2.00797800000000     13.43112100000000
H  -0.83640200000000      2.23577800000000     12.00402800000000
H   0.80170200000000     -2.26265900000000     11.99730900000000
H   0.18963000000000     -2.03785700000000     13.43414500000000
H  -2.00612200000000     -0.25664700000000     13.42965500000000
H  -2.25189000000000     -0.81135200000000     11.97094400000000
H   2.03307000000000      0.20290900000000     13.41620800000000
H   2.22615300000000      0.79372900000000     11.96595900000000
Ni  0.00846900000000     -0.00755500000000     16.64158200000000
O   0.03102800000000      2.07971000000000     16.63845000000000
O  -0.01409000000000     -2.09482100000000     16.64471400000000
O  -2.07877000000000      0.01502000000000     16.65269200000000
O   2.09570700000000     -0.03013000000000     16.63047200000000
O   0.01961200000000     -0.00454400000000     18.72894000000000
H   0.24618900000000      1.99654000000000     17.60551100000000
H   0.87753900000000      2.23823700000000     16.18025800000000
H  -0.20316400000000     -1.98539600000000     17.61641700000000
H  -0.87394900000000     -2.28246300000000     16.22544300000000
H  -2.24131200000000      0.73436000000000     17.29157100000000
H  -2.02179000000000      0.42803400000000     15.75703100000000
H   2.25299100000000     -0.89124500000000     16.20033700000000
H   2.01208600000000     -0.21473700000000     17.60427400000000
Ni  0.03075500000000     -0.00153200000000     20.81629800000000
O   0.05331400000000      2.08573400000000     20.81316700000000
O   0.00819600000000     -2.08879800000000     20.81943000000000
O  -2.05648300000000      0.02104300000000     20.82740799999999
O   2.11799300000000     -0.02410700000000     20.80518899999999
O   0.04189800000000      0.00148000000000     22.90365700000000
H  -0.13365900000000      1.97195000000000     21.78599100000000
H  -0.80056000000000      2.31563900000000     20.40355300000000
H   0.85286400000000     -2.26369000000000     20.36395100000000
H   0.22226100000000     -2.00639400000000     21.78700100000000
H  -2.27959700000000     -0.80626500000000     21.29138300000000
H  -1.94079800000000     -0.22395100000000     19.86854400000000
H   2.03665100000000      0.20156300000000     21.76970000000000
H   2.27999700000000      0.81572500000000     20.33572299999999
Ni  0.05304100000000      0.00449100000000     24.99101500000000
O   0.07560000000000      2.09175700000000     24.98788300000000
O   0.03048200000000     -2.08277400000000     24.99414600000000
O  -2.03419700000000      0.02706600000000     25.00212400000000
O   2.14027900000000     -0.01808400000000     24.97990500000000
O   0.06418400000000      0.00750300000000     27.07837299999999
H   0.33163700000000      2.00485700000000     25.94382400000000
H   0.90358100000000      2.23881800000000     24.49360600000000
H  -0.07789400000000     -1.95346600000000     25.97857899999999
H  -0.85649400000000     -2.29841500000000     24.65389900000000
H  -2.23130200000000      0.89705700000000     25.39449400000000
H  -1.90280800000000      0.20073400000000     24.02730700000000
H   2.21983900000000     -0.71421500000000     24.29704499999999
H   2.19233600000000     -0.46052300000000     25.85539600000000
```

```
Ni  0.07532700000000     0.01051500000000     29.16573100000000
O   0.09788600000000     2.09778000000000     29.16259900000000
O   0.05276800000000    -2.07675100000000     29.16886300000000
O  -2.01191100000000     0.03309000000000     29.17684100000000
O   2.16256600000000    -0.01206100000000     29.15462100000000
O   0.08647000000000     0.01352600000000     31.25308900000000
H  -0.07293000000000     2.05819600000000     30.13715000000000
H  -0.74399400000000     2.35381400000000     28.74525199999999
H  -0.80183200000000    -2.48598000000000     28.94747200000000
H   0.06413200000000    -1.93623400000000     30.15706000000000
H  -2.42682800000000    -0.76042400000000     29.55672300000000
H  -1.90522900000000    -0.12400800000000     28.19540399999999
H   2.41554200000000     0.89803800000000     29.39786599999999
H   1.98624600000000     0.02988300000000     28.17144599999999
Ni  0.09761300000000     0.01653800000000     33.34044700000000
O   0.12017200000000     2.10380300000000     33.33731500000000
O   0.07505400000000    -2.07072800000000     33.34357900000000
O  -1.98962500000000     0.03911300000000     33.35155700000000
O   2.18485200000000    -0.00603800000000     33.32933700000000
O   0.10875600000000     0.01955000000000     35.42780500000000
H   0.19155100000000     1.97000800000000     34.32004000000000
H  -0.80856900000000     2.36204400000000     33.19014400000000
H  -0.72673200000000    -2.53591000000000     33.63885400000000
H  -0.00348500000000    -1.93485800000000     32.35492300000000
H  -2.41188600000000    -0.78171500000000     33.65767100000000
H  -1.85160600000000    -0.04280100000000     32.36064700000000
H   2.42139600000000    -0.91894300000000     33.57767400000000
H   2.11417500000000    -0.01501600000000     32.33829400000000
Ni  0.11990000000000     0.02256300000000     37.51516300000000
O   0.14245800000000     2.10982900000000     37.51202900000000
O   0.09734100000000    -2.06470300000000     37.51829800000000
O  -1.96733900000000     0.04513800000000     37.52627700000000
O   2.20713800000000    -0.00001200000000     37.50405600000000
O   0.13104300000000     0.02557700000000     39.60252099999999
H   0.12540900000000     1.89931800000000     36.52886700000000
H  -0.62977000000000     2.67958800000000     37.68239200000000
H  -0.81795100000000    -2.36563700000000     37.67201299999999
H   0.16416100000000    -1.94158500000000     36.53238600000000
H  -2.47654000000000     0.80287400000000     37.86322900000000
H  -1.85644200000000     0.16481400000000     36.54112700000000
H   2.58636200000000     0.88609000000000     37.65710900000000
H   2.08783500000000    -0.04423100000000     36.51392000000000
H   0.61491500000000     0.77347200000000     39.99633099999998
H   0.59645200000000    -0.77319300000000     39.91338999999999
```

2. $[Fe(SCH_3)_4]^-$

A. Geometry

```
Fe   0.00000000000000    0.00000000000000    0.00000000000000
S   -1.34422876300000   -1.34426459700000    1.22915400000000
S    1.34426459700000   -1.34422876300000   -1.22915400000000
S    1.34422876300000    1.34426459700000    1.22915400000000
S   -1.34426459700000    1.34422876300000   -1.22915400000000
C   -0.23456088800000   -2.19578273700000    2.28657200000000
C    2.19578273700000   -0.23456088800000   -2.28657200000000
C    0.23456088800000    2.19578273700000    2.28657200000000
C   -2.19578273700000    0.23456088800000   -2.28657200000000
H    0.55144625600000   -2.50437101300000    1.78972600000000
H   -0.69929787200000   -2.92539324100000    2.71429100000000
H   -0.08168316200000   -1.55548165400000    3.09664700000000
H    2.50437101300000    0.55144625600000   -1.78972600000000
H    2.92539324100000   -0.69929787200000   -2.71429100000000
H    1.55548165400000   -0.08168316200000   -3.09664700000000
H   -0.55144625600000    2.50437101300000    1.78972600000000
H    0.69929787200000    2.92539324100000    2.71429100000000
H    0.08168316200000    1.55548165400000    3.09664700000000
H   -2.50437101300000   -0.55144625600000   -1.78972600000000
H   -2.92539324100000    0.69929787200000   -2.71429100000000
H   -1.55548165400000    0.08168316200000   -3.09664700000000
```

B. Branching diagrams

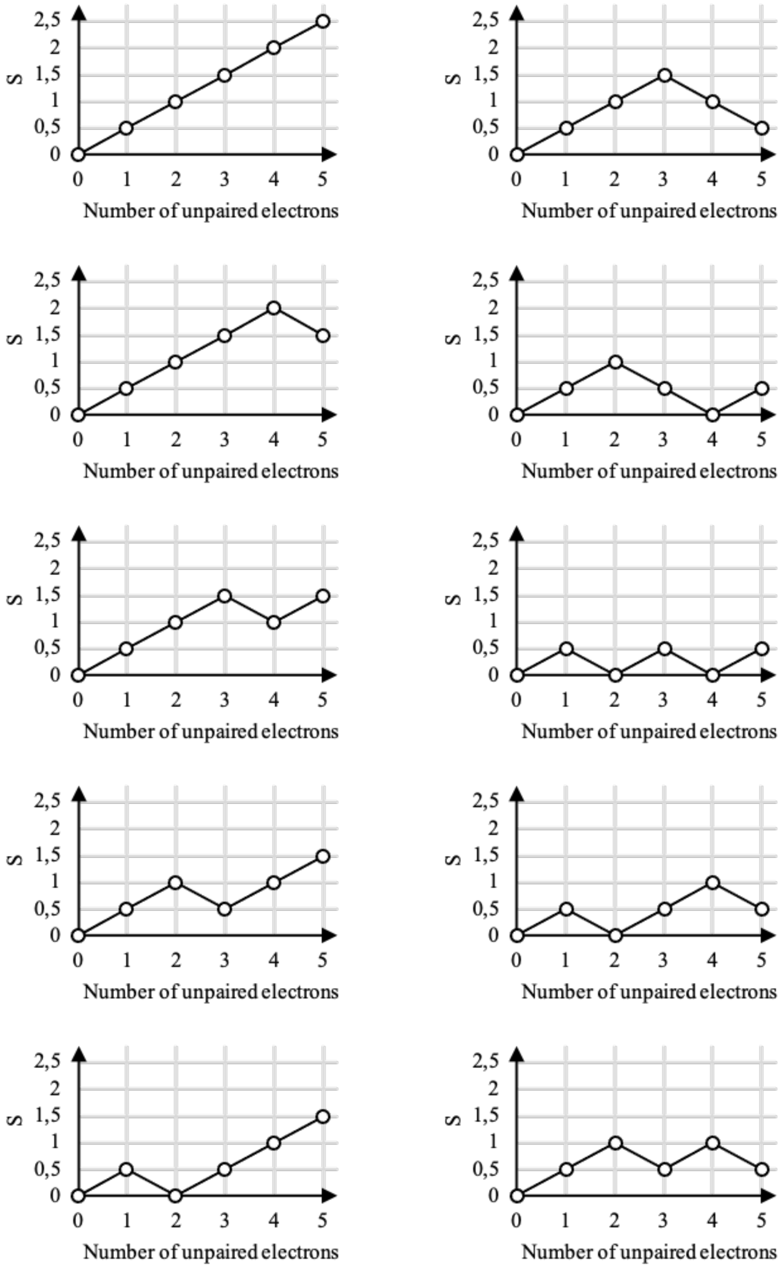

Figure S1. Branching diagrams for the sextet, quartet and doublet multiplicities of $[Fe(SCH_3)_4]^-$.

C. Sample Inputs

SAHF calculation for 5 electrons in 5 orbitals and $S = \frac{3}{2}$ of $[Fe(SCH_3)_4]^-$, using AVAS for the initial guess orbitals:

```
# [Fe(SCH3)4]- Quartet SAHF

! Def2-TZVP TightSCF

%pal
 nproc 8
end

%scf
 avas
  system
   norb 5
   nel 5
   center 0
   type d
  end
 end
 HFTyp ROHF
 ROHF_CASE SAHF
 ROHF_NumOp 2
 ROHF_NEL[1] 5
 ROHF_NORB[1] 5
end

*xyz -1 4
 Fe  0.00000000000000  0.00000000000000  0.00000000000000
 S  -1.34422876300000 -1.34426459700000  1.22915400000000
 S   1.34426459700000 -1.34422876300000 -1.22915400000000
 S   1.34422876300000  1.34426459700000  1.22915400000000
 S  -1.34426459700000  1.34422876300000 -1.22915400000000
 C  -0.23456088800000 -2.19578273700000  2.28657200000000
 C   2.19578273700000 -0.23456088800000 -2.28657200000000
 C   0.23456088800000  2.19578273700000  2.28657200000000
 C  -2.19578273700000  0.23456088800000 -2.28657200000000
 H   0.55144625600000 -2.50437101300000  1.78972600000000
 H  -0.69929787200000 -2.92539324100000  2.71429100000000
 H  -0.08168316200000 -1.55548165400000  3.09664700000000
 H   2.50437101300000  0.55144625600000 -1.78972600000000
 H   2.92539324100000 -0.69929787200000 -2.71429100000000
 H   1.55548165400000 -0.08168316200000 -3.09664700000000
 H  -0.55144625600000  2.50437101300000  1.78972600000000
 H   0.69929787200000  2.92539324100000  2.71429100000000
 H   0.08168316200000  1.55548165400000  3.09664700000000
 H  -2.50437101300000 -0.55144625600000 -1.78972600000000
 H  -2.92539324100000  0.69929787200000 -2.71429100000000
 H  -1.55548165400000  0.08168316200000 -3.09664700000000
end
```

CSF-ROHF calculation for the CSF $[++-++]$ with $S=\frac{3}{2}$ of $[Fe(SCH_3)_4]^-$, using the converged SAHF orbitals as initial guess:

```
# [Fe(SCH3)4]- Quartet ROHF [++-++] CSF

! Def2-TZVP TightSCF MOREAD

%moinp "fesch3_quart_sahf.gbw"

%pal
 nproc 8
end

%scf
 HFTyp ROHF
 ROHF_CASE USER_CSF
 ROHF_REF {1 1 -1 1 1} end
 MaxIter 1000
end

*xyz -1 4
 Fe  0.00000000000000  0.00000000000000  0.00000000000000
 S  -1.34422876300000 -1.34426459700000  1.22915400000000
 S   1.34426459700000 -1.34422876300000 -1.22915400000000
 S   1.34422876300000  1.34426459700000  1.22915400000000
 S  -1.34426459700000  1.34422876300000 -1.22915400000000
 C  -0.23456088800000 -2.19578273700000  2.28657200000000
 C   2.19578273700000 -0.23456088800000 -2.28657200000000
 C   0.23456088800000  2.19578273700000  2.28657200000000
 C  -2.19578273700000  0.23456088800000 -2.28657200000000
 H   0.55144625600000 -2.50437101300000  1.78972600000000
 H  -0.69929787200000 -2.92539324100000  2.71429100000000
 H  -0.08168316200000 -1.55548165400000  3.09664700000000
 H   2.50437101300000  0.55144625600000 -1.78972600000000
 H   2.92539324100000 -0.69929787200000 -2.71429100000000
 H   1.55548165400000 -0.08168316200000 -3.09664700000000
 H  -0.55144625600000  2.50437101300000  1.78972600000000
 H   0.69929787200000  2.92539324100000  2.71429100000000
 H   0.08168316200000  1.55548165400000  3.09664700000000
 H  -2.50437101300000 -0.55144625600000 -1.78972600000000
 H  -2.92539324100000  0.69929787200000 -2.71429100000000
 H  -1.55548165400000  0.08168316200000 -3.09664700000000
end
```

3. $[Fe_2S_2(SCH_3)_4]^{2-}$

A. Geometry

```
Fe  0.00000000000000      0.00000000000000     -1.34356781200000
Fe  0.00000000000000      0.00000000000000      1.34356781200000
S   1.07173350100000      1.37336608200000      0.00000000000000
S   1.34671428400000     -1.34590148600000     -2.65162144900000
S  -1.34671428400000      1.34590148600000     -2.65162144900000
S  -1.07173350100000     -1.37336608200000      0.00000000000000
S  -1.34671428400000      1.34590148600000      2.65162144900000
S   1.34671428400000     -1.34590148600000      2.65162144900000
C  -2.48566330400000      0.36254339300000     -3.60079527600000
H  -3.31993751600000      0.59673175500000     -3.50588279500000
H  -2.34744650700000      0.38829290300000     -4.46338059000000
H  -2.47240470900000     -0.48571120300000     -3.40416734300000
C   2.48566330400000     -0.36254339300000     -3.60079527600000
H   3.31993751600000     -0.59673175500000     -3.50588279500000
H   2.34744650700000     -0.38829290300000     -4.46338059000000
H   2.47240470900000      0.48571120300000     -3.40416734300000
C   2.48566330400000     -0.36254339300000      3.60079527600000
H   2.34744650700000     -0.38829290300000      4.46338059000000
H   3.31993751600000     -0.59673175500000      3.50588279500000
H   2.47240470900000      0.48571120300000      3.40416734300000
C  -2.48566330400000      0.36254339300000      3.60079527600000
H  -3.31993751600000      0.59673175500000      3.50588279500000
H  -2.47240470900000     -0.48571120300000      3.40416734300000
H  -2.34744650700000      0.38829290300000      4.46338059000000
```

B. Sample Inputs

SAHF calculation for 10 electrons in 10 orbitals and $S = 0$ of $[Fe_2S_2(SCH_3)_4]^{2-}$, using AVAS for the initial guess orbitals:

```
# [Fe2S2(SCH3)4]2- SAHF with AVAS Guess Orbitals

! Def2-TZVP TightSCF

%pal
 nproc 8
end

%scf
 avas
  system
   nel 10
   norb 10
   center 0,1
   type d,d
  end
 end
 HFTyp ROHF
 ROHF_Case SAHF
 ROHF_NumOp 2
 ROHF_NEL[1] 10
 ROHF_NORB[1] 10
end

*xyz -2 1
 Fe   0.000000000   0.000000000  -1.343567812
 Fe   0.000000000   0.000000000   1.343567812
 S    1.071733501   1.373366082   0.000000000
 S    1.346714284  -1.345901486  -2.651621449
 S   -1.346714284   1.345901486  -2.651621449
 S   -1.071733501  -1.373366082   0.000000000
 S   -1.346714284   1.345901486   2.651621449
 S    1.346714284  -1.345901486   2.651621449
 C   -2.485663304   0.362543393  -3.600795276
 H   -3.319937516   0.596731755  -3.505882795
 H   -2.347446507   0.388292903  -4.463380590
 H   -2.472404709  -0.485711203  -3.404167343
 C    2.485663304  -0.362543393  -3.600795276
 H    3.319937516  -0.596731755  -3.505882795
 H    2.347446507  -0.388292903  -4.463380590
 H    2.472404709   0.485711203  -3.404167343
 C    2.485663304  -0.362543393   3.600795276
 H    2.347446507  -0.388292903   4.463380590
 H    3.319937516  -0.596731755   3.505882795
 H    2.472404709   0.485711203   3.404167343
 C   -2.485663304   0.362543393   3.600795276
 H   -3.319937516   0.596731755   3.505882795
 H   -2.472404709  -0.485711203   3.404167343
 H   -2.347446507   0.388292903   4.463380590
end
```

CSF-ROHF calculation for the CSF $[+++++-----]$ with $S = 0$ of $[Fe_2S_2(SCH_3)_4]^{2-}$, using the converged SAHF orbitals as initial guess:

```
# [Fe2S2(SCH3)4]2- AF-ROHF with localized SAHF orbitals

! Def2-TZVP TightSCF MOREAD

%moinp "fe2sch_sahf.loc"

%pal
 nproc 8
end

%scf
 Rotate {88,94,90} {92,96,90} end
 HFTyp ROHF
 ROHF_Case AF_CSF
 ROHF_AFORBS 5,5
 MaxIter 500
end

*xyz -2 1
 Fe   0.000000000   0.000000000  -1.343567812
 Fe   0.000000000   0.000000000   1.343567812
 S    1.071733501   1.373366082   0.000000000
 S    1.346714284  -1.345901486  -2.651621449
 S   -1.346714284   1.345901486  -2.651621449
 S   -1.071733501  -1.373366082   0.000000000
 S   -1.346714284   1.345901486   2.651621449
 S    1.346714284  -1.345901486   2.651621449
 C   -2.485663304   0.362543393  -3.600795276
 H   -3.319937516   0.596731755  -3.505882795
 H   -2.347446507   0.388292903  -4.463380590
 H   -2.472404709  -0.485711203  -3.404167343
 C    2.485663304  -0.362543393  -3.600795276
 H    3.319937516  -0.596731755  -3.505882795
 H    2.347446507  -0.388292903  -4.463380590
 H    2.472404709   0.485711203  -3.404167343
 C    2.485663304  -0.362543393   3.600795276
 H    2.347446507  -0.388292903   4.463380590
 H    3.319937516  -0.596731755   3.505882795
 H    2.472404709   0.485711203   3.404167343
 C   -2.485663304   0.362543393   3.600795276
 H   -3.319937516   0.596731755   3.505882795
 H   -2.472404709  -0.485711203   3.404167343
 H   -2.347446507   0.388292903   4.463380590
end
```

4. $[Gd_2Cl_{11}]^{5-}$

A. Geometry

```
Cl  2.82651000000000      0.00000000000000      0.00000000000000
Cl  0.00000000000000      2.82651000000000      0.00000000000000
Cl  0.00000000000000     -2.82651000000000     -0.00000000000000
Cl  0.00000000000000      0.00000000000000     -2.82651000000000
Cl  0.00000000000000     -0.00000000000000      2.82651000000000
Cl -2.82651000000000      0.00000000000000      0.00000000000000
Cl -5.65302000000000      2.82651000000000      0.00000000000000
Cl -5.65302000000000     -2.82651000000000     -0.00000000000000
Cl -5.65302000000000      0.00000000000000     -2.82651000000000
Cl -5.65302000000000      0.00000000000000      2.82651000000000
Cl -8.47953000000000      0.00000000000000      0.00000000000000
Gd  0.00000000000000      0.00000000000000      0.00000000000000
Gd -5.65302000000000      0.00000000000000      0.00000000000000
```

B. Molecular Orbitals

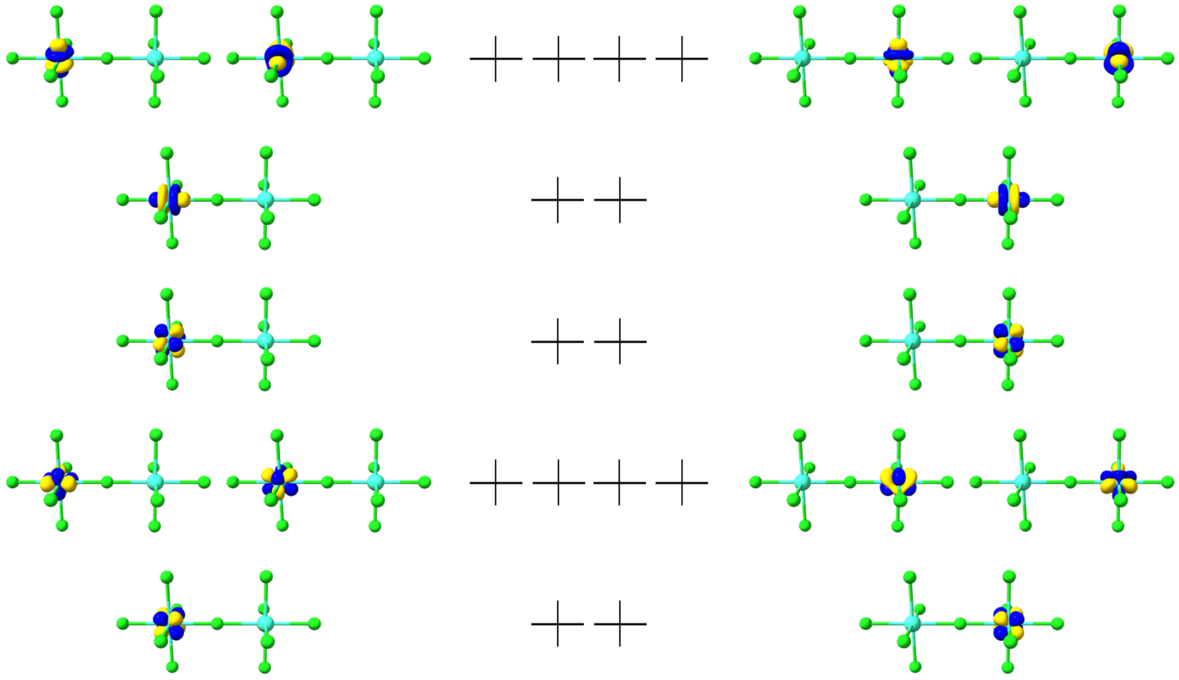

Figure S2. CSF-ROHF molecular orbitals for the CSF [+ + + + + + + − − − − − − −] of $[Gd_2Cl_{11}]^{5-}$.

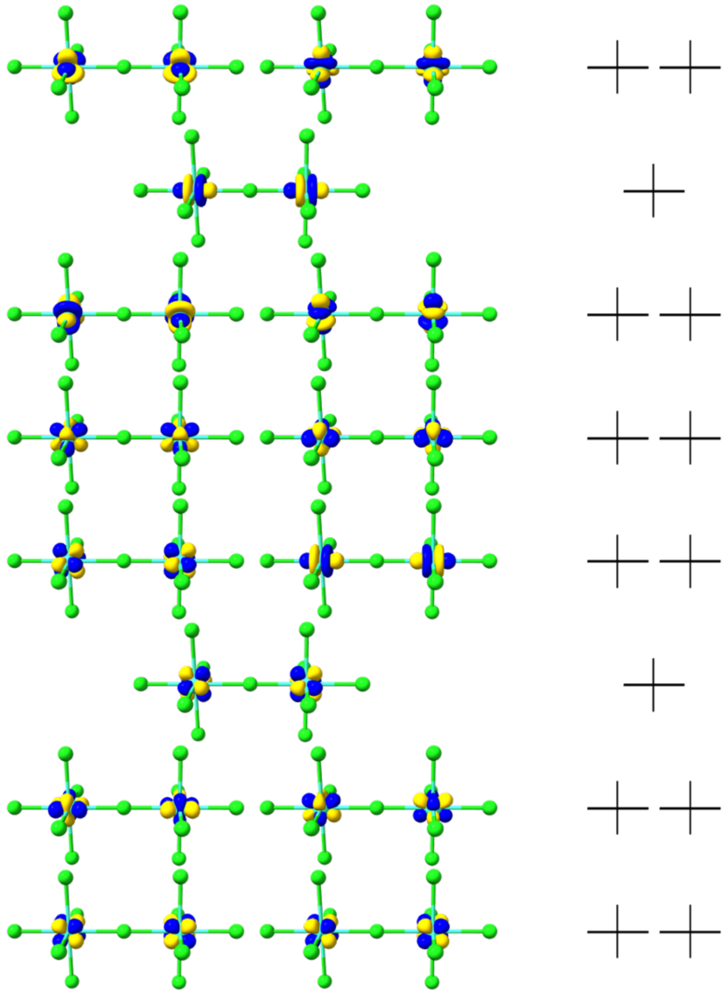

Figure S3. HS-ROHF molecular orbitals for $[Gd_2Cl_{11}]^{5-}$.

5. $[Fe_4S_4(SCH_3)_4]^{2-}$

A. Geometry

```
S   0.04000000000000    -1.78000000000000    -1.29000000000000
S  -0.04000000000000     1.78000000000000    -1.29000000000000
S   1.78000000000000    -0.04000000000000     1.29000000000000
S  -1.78000000000000     0.04000000000000     1.29000000000000
Fe  0.05000000000000    -1.37000000000000     1.01000000000000
Fe -1.38000000000000     0.05000000000000    -1.00000000000000
Fe -0.05000000000000     1.38000000000000     1.00000000000000
Fe  1.37000000000000    -0.05000000000000    -1.01000000000000
S   0.24000000000000     3.30000000000000     2.14000000000000
S  -0.24000000000000    -3.29000000000000     2.14000000000000
S  -3.29000000000000    -0.24000000000000    -2.14000000000000
S   3.29000000000000     0.24000000000000    -2.14000000000000
C  -3.80000000000000    -1.84000000000000    -1.38000000000000
H  -3.91000000000000    -1.71000000000000    -0.29000000000000
H  -4.76000000000000    -2.17000000000000    -1.81000000000000
H  -3.03000000000000    -2.60000000000000    -1.56000000000000
C   3.80000000000000     1.83000000000000    -1.38000000000000
H   3.91000000000000     1.71000000000000    -0.29000000000000
H   4.76000000000000     2.16000000000000    -1.81000000000000
H   3.03000000000000     2.59000000000000    -1.55000000000000
C  -1.83000000000000    -3.80000000000000     1.38000000000000
H  -2.16000000000000    -4.76000000000000     1.81000000000000
H  -2.59000000000000    -3.03000000000000     1.55000000000000
H  -1.70000000000000    -3.91000000000000     0.29000000000000
C   1.84000000000000     3.80000000000000     1.38000000000000
H   2.17000000000000     4.76000000000000     1.81000000000000
H   2.60000000000000     3.03000000000000     1.56000000000000
H   1.71000000000000     3.91000000000000     0.29000000000000
```

6. $[Cu_3(OH)_3(en)_3]^{3+}$

A. Geometry

```
Cu  0.07390700000000     1.73092000000000     0.01728100000000
Cu  1.81809500000000    -1.29635400000000    -0.06114800000000
Cu -1.76965100000000    -1.21814100000000     0.07267000000000
O  -1.11343200000000     0.44627400000000     0.92915300000000
O   1.23194000000000     0.41405300000000    -0.88164600000000
O   0.00802500000000    -2.01785300000000    -0.04229800000000
H  -0.67662500000000     0.15321200000000     1.75194100000000
H   0.84086100000000     0.17106800000000    -1.74256200000000
H  -0.01157100000000    -2.97021000000000     0.15205400000000
N  -0.62089900000000     3.27656000000000     1.18293300000000
N   0.82029800000000     3.26233500000000    -1.13975700000000
C  -0.52146500000000     4.56387700000000     0.42541700000000
H  -1.58160500000000     3.09354300000000     1.48805800000000
C   0.77778000000000     4.54127000000000    -0.36247200000000
H  -0.56199500000000     5.43010500000000     1.10083200000000
H  -1.38740900000000     4.62578800000000    -0.24858200000000
H   1.77207700000000     3.04496800000000    -1.45056000000000
H   1.64541000000000     4.55438200000000     0.31203600000000
H   0.85628700000000     5.41479800000000    -1.02501300000000
H   0.25669200000000     3.35170300000000    -1.99421900000000
H  -0.05715100000000     3.32866500000000     2.04053600000000
N   3.76166100000000    -0.70464200000000    -0.29823400000000
N   2.69106900000000    -2.94869100000000     0.83010000000000
C   4.12438700000000    -2.63407000000000     1.13987200000000
H   2.20605000000000    -3.26122700000000     1.67785100000000
C   4.68937700000000    -1.84646900000000    -0.02897100000000
H   4.70222700000000    -3.55162700000000     1.32005400000000
H   4.14384100000000    -2.03907700000000     2.06385900000000
H   4.73442000000000    -2.46528500000000    -0.93626000000000
H   5.70837200000000    -1.49381700000000     0.18460900000000
H   3.89700700000000    -0.32506600000000    -1.24067700000000
H   3.94736000000000     0.07036700000000     0.34860100000000
H   2.64871500000000    -3.73410900000000     0.16832600000000
N  -2.72036100000000    -2.88368800000000    -0.70205800000000
N  -3.66319300000000    -0.44825300000000     0.09463100000000
C  -4.66373900000000    -1.54643800000000    -0.08398300000000
H  -3.81394600000000     0.06029000000000     0.97155200000000
C  -4.10940100000000    -2.50669200000000    -1.12204100000000
H  -5.64255200000000    -1.15021300000000    -0.38851000000000
H  -4.79163500000000    -2.04603500000000     0.88649400000000
H  -4.04250600000000    -2.02529900000000    -2.10774600000000
H  -4.74848100000000    -3.39476100000000    -1.22706700000000
H  -2.20327300000000    -3.28730700000000    -1.49016700000000
H  -2.76412000000000    -3.61553600000000     0.01812800000000
H  -3.75470400000000     0.24348600000000    -0.65837700000000
```

7. [Co(^1L_N)$_2$]

A. Geometry

```
Co  0.19114500000000   -0.17442700000000   -0.06452000000000
N   1.55368800000000    1.05392800000000   -0.06859800000000
N   1.55831300000000   -1.39562900000000   -0.13419400000000
N  -1.17597600000000    1.04675200000000    0.00680900000000
N  -1.17128200000000   -1.40292200000000   -0.05634000000000
C   2.82159500000000   -0.88598400000000   -0.15190100000000
H   1.49470000000000   -2.41478500000000   -0.15872200000000
C   2.81865900000000    0.55037000000000   -0.11335200000000
C   4.03879100000000    1.25450100000000   -0.12470900000000
C   4.04451700000000   -1.58355000000000   -0.20029800000000
C   5.23765700000000   -0.86651400000000   -0.21031000000000
C   5.23482300000000    0.54392300000000   -0.17277100000000
C  -2.43909900000000    0.53691400000000    0.03167800000000
H  -1.11233700000000    2.06589900000000    0.03213300000000
C  -2.43609700000000   -0.89954800000000   -0.00493100000000
C  -3.65606000000000   -1.60389300000000    0.01389000000000
C  -4.85196200000000   -0.89344700000000    0.06773700000000
C  -4.85485600000000    0.51707400000000    0.10352800000000
C  -3.66189700000000    1.23433000000000    0.08609200000000
H  -5.80501200000000    1.05123300000000    0.14563700000000
H  -3.66543000000000    2.32626900000000    0.11374600000000
H  -3.65494800000000   -2.69584600000000   -0.01371600000000
H  -5.79991900000000   -1.43294400000000    0.08247400000000
H   6.18291400000000    1.08325500000000   -0.18162400000000
H   4.03773600000000    2.34638000000000   -0.09539200000000
H   4.04801700000000   -2.67541700000000   -0.22961200000000
H   6.18790900000000   -1.40080600000000   -0.24754300000000
H  -1.10390200000000   -2.42178200000000   -0.08253700000000
H   1.48644500000000    2.07268600000000   -0.03874500000000
```
